# PrimPol prevents APOBEC/AID family mediated DNA mutagenesis

**DOI:** 10.1093/nar/gkw123

**Published:** 2016-02-28

**Authors:** Bas Pilzecker, Olimpia Alessandra Buoninfante, Colin Pritchard, Olga S. Blomberg, Ivo J. Huijbers, Paul C.M. van den Berk, Heinz Jacobs

**Affiliations:** 1 Division of Biological Stress Response, The Netherlands Cancer Institute, Plesmanlaan 121, 1066 CX Amsterdam, The Netherlands; 2 Mouse Clinic for Cancer and Aging research (MCCA) Transgenic Facility, The Netherlands Cancer Institute, Plesmanlaan 121, 1066 CX Amsterdam, The Netherlands

## Abstract

PrimPol is a DNA damage tolerant polymerase displaying both translesion synthesis (TLS) and (re)-priming properties. This led us to study the consequences of a *PrimPol* deficiency in tolerating mutagenic lesions induced by members of the APOBEC/AID family of cytosine deaminases. Interestingly, during somatic hypermutation, PrimPol counteracts the generation of C>G transversions on the leading strand. Independently, mutation analyses in human invasive breast cancer confirmed a pro-mutagenic activity of APOBEC3B and revealed a genome-wide anti-mutagenic activity of PRIMPOL as well as most Y-family TLS polymerases. PRIMPOL especially prevents APOBEC3B targeted cytosine mutations within TpC dinucleotides. As C transversions induced by APOBEC/AID family members depend on the formation of AP-sites, we propose that PrimPol reprimes preferentially downstream of AP-sites on the leading strand, to prohibit error-prone TLS and simultaneously stimulate error-free homology directed repair. These *in vivo* studies are the first demonstrating a critical anti-mutagenic activity of PrimPol in genome maintenance.

## INTRODUCTION

DNA damage tolerance (DDT) is an integral part of the DNA damage response network that maintains the integrity of the genome (1–4). DDT enables replication to continue in the presence of a fork-stalling lesion. Principally, four distinct DDT pathways could be distinguished, (i) direct translesion synthesis (TLS) across the damaged template (‘on the flight’), (ii) repriming behind the damaged template followed by gap filling TLS (post-replicative TLS), (iii) direct template switching (TS), taking advantage of the intact template of the sister chromatid or (iv) repriming behind the damaged template, where the remaining gap is restored by homology directed repair. TLS enables direct replicative bypass of lesions that otherwise stall the replicative DNA polymerases. It involves a set of specialized proofread inactive TLS polymerases that can accommodate non-Watson/Crick base pairs in their catalytic centre. This capacity can lead to misinsertion errors and renders TLS potentially mutagenic. In contrast, TS avoids the damage by taking advantage of the intact template on the sister chromatid and therefore is relatively error-free. Repriming downstream of the lesion is another elegant way to relief acute replication stress. Common to all DDT pathways, they enable replication progression in the presence of otherwise replication blocking lesions and in this way contribute to genome stability by prevention replication fork collapses (1–4).

The capacity to tolerate lesions in the DNA template is essential for programmed mutation pathways that operate in higher eukaryotes. These intentional mutation processes are initiated by cytosine (C) deaminases of the APOBEC/AID family. Members of this family deaminate C to uracil (U) in RNA and single-stranded DNA. Replication over U generates C>T transitions. Alternatively, uracil glycosylases can process Us into non-instructive AP-sites, which upon TLS can give rise to both C transitions (C>T) and transversions (C>G, C>A). In the innate immune system cytosine deamination by APOBEC3A and APOBEC3B can mutagenize and inactivate retro-elements. In the adaptive antibody-dependent immune system, cytosine deamination by the Activation-Induced cytidine Deaminase (AID) induces somatic hypermutation (SHM) and class switch recombination (CSR) of Ig genes, enabling the generation of high affinity antibody variants (SHM) with new effector functions and tissue distribution (CSR).

SHM is triggered in antigen activated, centroblastic B cells of the germinal center, where the expression of AID becomes transiently induced (5–9). SHM is confined to the hypermutation domain, the transcribed regions encompassing the VDJ and VJ exon of rearranged Ig heavy and light chain genes, respectively (7–9). Mutations that increase the affinity between the clonotypic surface Ig and cognate antigen provide a selective advantage to the B cell. As a consequence high affinity antibodies dominate immune recall responses, a phenomenon known as antibody affinity maturation.

CSR is a programmed recombination process between two active switch regions of the *Igh* locus that enables antigen activated B cells to switch their IgH isotype and hence change the antibody effector function and tissue distribution (10,11).

Other members of the APOBEC/AID family have been implicated in establishing innate immunity, specifically by controlling retro-elements. For example, APOBEC3A and APOBEC3B can inactivate retroviruses by inducing predominantly G/C>A/T transitions *in vitro* as well *in vivo* (12). Aberrant targeting of APOBEC3B has been linked to active genome wide mutagenesis as well as kataegis in various cancers (13,14). Especially, breast cancer, cervical cancer, bladder cancer, lung squamous cell carcinoma, lung adenocarcinoma, and head and neck cancer are characterized by a high mutation load of cytosines at TpC dinucleotides, the preferred target sequence of APOBEC3A/B. In breast cancer specifically APOBEC3B has been identified as highly mutagenic (14–22).

PrimPol has recently been identified as a novel and unique DNA polymerase, which *in vitro* displays both primase and TLS activity (23–26). *In vitro*, it has been demonstrated that PrimPol cannot incorporate nucleotides across an AP site but potentially can reprime downstream of a replication-blocking lesion under nuclear like conditions. Alternatively, in the presence of high manganese ion concentration, a condition more closely resembling mitochondria, PrimPol can generate single nucleotide deletion in the presence of a template AP-site (25). In addition, like most DNA polymerases, PrimPol incorporates an A opposite a template U (23,26). Given the contribution of TLS polymerases in establishing somatic mutations at non-instructive AP-sites, we wondered whether PrimPol modulates APOBEC/AID-induced mutagenesis. This study focuses in particular on the *in vivo* role of PrimPol in establishing the characteristic somatic mutation spectra downstream of APOBEC/AID family member induced AP-sites in murine SHM and human invasive breast cancer. Our data reveal a strand-biased anti-mutagenic activity of PrimPol, where PrimPol reprimes efficiently downstream of AP-sites on the leading strand, thereby contributing to replication fork progression and simultaneously preventing error-prone TLS.

## MATERIALS AND METHODS

### Generation of *PrimPol* mouse

Zygotes isolated from C57BL/6N mice were co-injected with *in vitro* transcribed CRISPR/Cas9 mRNA and sgRNA (sequence TTAACAAATTGGCCAACCC-AGG, designed using the crispr.mit.edu tool) from pX330 plasmid (27). This sgRNA targets CRISPR/Cas9 to exon 5 of the *PrimPol* locus (ENSMUSE00001229433). Offspring was tested by PCR for *PrimPol* inactivating mutations using the primers (FW: GTC GGA CAA CCT AGG TTC AGT GC, Rev: CCC TGA ATT TCA TCT CAT TGT CTA C) and ABI^®^ sequencing (3730 DNA analyzer, Applied Biosystems). A *PrimPol* mutant, carrying a 4-bp CCAA deletion in exon 5 was selected, backcrossed once onto C57BL/6J and maintained heterozygous. For genotyping the wild-type and mutant alleles are distinguished by MscI digestion, where the non-digested product is indicative for a mutant allele. Experiments were approved by an independent animal ethics committee of the Netherlands Cancer Institute (Amsterdam, Netherlands) (DEC number 13007) and executed according national and European guidelines.

### cDNA synthesis and qPCR

Total RNA was isolated from wild-type and PrimPol^Δ/Δ^ mouse embryonic fibroblasts (MEFs) using RNeasy mini (Qiagen). cDNA library was synthesized by Invitrogen Superscript III kit and oligo dT primers. A nested PCR was performed on the cDNA spanning exon 1–15 (fwd GCT CTG GTT CCC GCC ATC TCT, rev CTT TCT CTC CAG GCT CTG GGA CA) followed by a nested primer set spanning exon 3–14 (fwd TGG CCA AGC CAG AAG AAC CAT CCT, rev CGT CAT CCC AGG CAG CGG CA). Subsequently, the cDNA was cut using MscI enzyme (Thermo Scientific), verifing the CCAA deletion. The deletion removed the MscI site in the wild-type allele.

For qPCR total RNA was isolated from wild-type and PrimPol^Δ/Δ^ MEFs using RNeasy mini (Qiagen). cDNA library was synthesized using Invitrogen Superscript III kit and random hexamer oligos. qPCR was performed with Fast SYBR® Green Master Mix (Thermo Scientific) and the LightCycler 480II (Roche). Normalization was performed to GAPDH (fwd: CAA TGA CCC CTT CAT TGA CC, rev: GAT CTC GCT CCT GGA AGA TG). PrimPol mRNA was amplified with primers specific for the exon-junction 4–5 (fwd: GAG TGC AAA AGG GGA AAT GG, rev: ATA ACT TCA TAG CAG TGC AAG AG) and the exon-junction 8–9 (fwd: CTA TCT TCC CTG GTG AGC AAT, rev: CTG AAG TGC CAG TAC TGT TAA A).

### Derivation of primary pre-B cell cultures and mouse embryonic fibroblast cell lines

E14.5 embryos were isolated from intercrosses of heterozygous *PrimPol* mice. Single cell suspensions were generated from fetal livers and subsequently cultured on ST2 feeder cells in IL7-containing complete medium (Iscoves, 8% FCS and penicillin/streptomycin) for the generation of pre-B cell cultures. MEFs were isolated using Trypsin and cell strainers, according to (28,29). Primary MEF cultures were established under low oxygen (3%) conditions. MEFs were immortalized (2 per genotype) using lentiviral expression of a p53-specific shRNA (30). Immortalized MEFs were grown in complete IMDM medium and incubated at 37°C in 5% CO_2_ and normal oxygen levels.

### Flow cytometry

Lymphoid cells from bone marrow, thymus and spleen were isolated from 8-week old mice. To reveal T cell progenitor subsets in the thymus, single cell suspensions were stained with conjugated antibodies, specific for CD3-FITC (Biolegend), CD4-APC, CD8a-PerCp-Cy5.5, CD25-PE, CD44-APCCy7 (Biolegend), TCRβ-Pacific Blue (Biolegend). To analyse lymphoid subsets in the spleen, single cell suspensions were stained with CD3-FITC, CD4-APC, CD8a-PerCp-Cy5.5, CD19-APCH7, CD45R(B220)-PacificBlue, IgD-PE (eBioscience), IgM-PECy7 (eBioscience). To define the B cell progenitor subsets, suspensions from the bone marrow were stained with IgD-FITC, CD25-PE, IgM-PECy7 (eBioscience), CD45R (B220)-PacificBlue, CD117 (cKit)-APC (eBioscience), CD19-APCH7. Dead cells were excluded from the analysis by propidium iodide staining. Antibodies were purchased from BD Pharmingen unless mentioned otherwise. Samples were measured on a FACS Fortessa^®^ and analysed using FlowJo^®^ software (Version: 10.0.8r1).

### Class switch recombination

Single cells suspensions were prepared from the spleens of 8-week old *PrimPol^Δ/Δ^* and their wild-type littermates. After the lysis of red blood cells, naïve splenic B cells were enriched by CD43 depletion using biotinylated anti-CD43 (Clone S7, BD Biosciences), and the IMag^®^ system (BD Biosciences), as described by the manufacturer. Purified B cells were labelled for 10 min at 37°C with 5 μM carboxy fluorescein diacetate succinimidyl ester (CFSE, Molecular Probes) in IMDM medium containing 2% FCS. After washing, cells were cultured in IMDM, supplemented with 8% FCS, 50 μM 2-mercapthoethanol, penicillin/streptomycin at a density of 10^5^ cells/well in 24 well plates. CSR to IgG3 and IgG1 was induced by exposure to LPS (50 μg/ml *Escherichia Coli* LPS, 055:B5, Sigma) or LPS/rIL-4 (rIL4 20 ng/ml). Four days later, the proliferative capacity was determined by CFSE dilution; CSR frequencies were determined by staining with CD19-PercpCy5.5 (BD), IgM-APC, and IgG3-PE (LPS cultures) or IgG1-PE (LPS/rIL4 cultures). IgM-APC, IgG1-PE and IgG3-PE were purchased from Southern Biotech. Data were acquired by flow cytometry (Fortessa^®^, BD) and analysed using FlowJo software (Version: 10.0.8r1).

### Somatic hypermutation and statistics

Viable (DAPI^−^) germinal centre B cells (CD19-APC^+^, PNA^high^-FITC, CD95-PE^+^) were sorted from Peyer's patches of 8-week old *PrimPol^Δ/Δ^* and their wild-type littermates on an Aria^®^ sorter (Becton Dickinson). DNA was extracted using proteinase K treatment and ethanol precipitation. The J_H_4 intronic sequence of rearranged VHJ558 family members were amplified by PCR using Pfu Ultra^®^ polymerase (Stratagene) (31). PCR products were purified using the QIAquick^®^ Gel Extraction kit (Qiagen), cloned into the TOPO II blunt vector (Invitrogen Life Technologies). Plasmid DNA was isolated using High Pure Plasmid kit (Roche) and sequenced on a 3730 DNA analyzer (Applied Biosystems). Sequence alignments were performed using Seqman software (DNAStar version 12,2,0).

Calculations exclude non-mutated sequences, insertions and deletions. Clonally related sequences with identical mutations and complementary determining region 3 and duplicated sequences were counted only once. Statistic analysis of the mutation spectra was performed using the χ^2^-test, with the number of mutations of base x and number sequenced base x. For the corrected percentage, the number of mutations were adjusted such that each base contributes 25% of the sequence. Mutation frequencies are expressed as the percentage of a defined nucleotide substitution at base (X) relative to all sequenced bases (X).

### DNA fibre analysis

Per well of a 6-well plate, 7.5 × 10^4^ MEFs were seeded and cultured overnight in IMDM medium. Prior to UVC exposure (20 or 40 J/m^2^), MEFs were incubated in medium containing 25 μM 5-Chloro-2′-deoxyuridine (CldU) for 20 min at 37°C. After UVC exposure, medium containing 500 μM 5-Iodo-2′-deoxyuridine (IdU) was added, resulting in a final concentration of 250 μM IdU and 12.5 μM CldU. After 20 min at 37°C, cells were trypsinized, 2 μl of a suspension of 3 × 10^5^ MEFs/ml was spotted onto a microscope slide, incubated for 5 min and lysed with 7 μl lysis buffer (200 mM Tris-HCl pH7.4, 50 mM EDTA, 0.5% SDS) for 3 min. Slides were tilted to 15°C to allow the DNA to run down the slide. Next, slides were air dried and subsequently fixed in methanol-acetic acid (3:1). After rehydration, fixed DNA fibres were denatured in 2.5M HCl for 75 min. Incorporation of CldU was detected using rat-α-BrdU antibodies (1:500; BU1/75, AbD Serotec) and Alexafluor-555-labelled goat-α-rat antibodies (1:500; Molecular Probes), whereas incorporated IdU was detected using mouse-α-BrdU antibodies (1:750; Clone B44, BD) and Alexafluor-488-labelled goat-α-mouse antibodies (1:500; Molecular Probes). Finally, slides were mounted in Fluoro-Gel (Electron Microscopy Sciences). Microscopy was performed using a fluorescent microscope (Zeiss Axiovert S 100).

### Pre-B cell survival assay

2 × 10^4^ pre-B cells were seeded on ST2 feeder cells in 24 well plates in 0.5 ml complete medium and IL7 prior to UV-C irradiation (254 nm, UVC irradiation chamber, Dr Gröbel UV-Elektronik, GmbH, Germany). After 15 min, cells were irradiated and cultured in 1 ml complete medium and IL7. For determining the survival, pre-B cells were harvested after 3 days of culture and live (propidium iodine negative) pre-B cells were counted by on a FACSArray (Becton Dickinson). Data were analysed using FlowJo software.

### Mutation and gene expression analysis of TCGA invasive breast cancer (BRCA)

To analyse the impact of PrimPol expression on the mutation load, we mined the mutation and gene expression data from The Cancer Genome Atlas (TCGA). Normalized RNAseqV2 containing RNA expression and level-2 exome mutation data from invasive breast cancers (BRCA) were retrieved and analysed in R using R Studio (Version 0.98.983). Duplicates in the data sets were taken out. For statistical analyses of the mutational frequencies between all tumours and *PRIMPOL* homozygous deleted tumours we applied the Mann-Whitney test. To correlate the mutation load of each tumour to the level of gene expression we applied the Spearman's correlation.

For mutational analysis of BRCA, the sense (C>G) and antisense (G>C) mutations were taken together, since there was no difference detected in total numbers.

## RESULTS

### CRISPR/Cas9 mediated inactivation of PrimPol in the mouse germline

**Figure 1.**
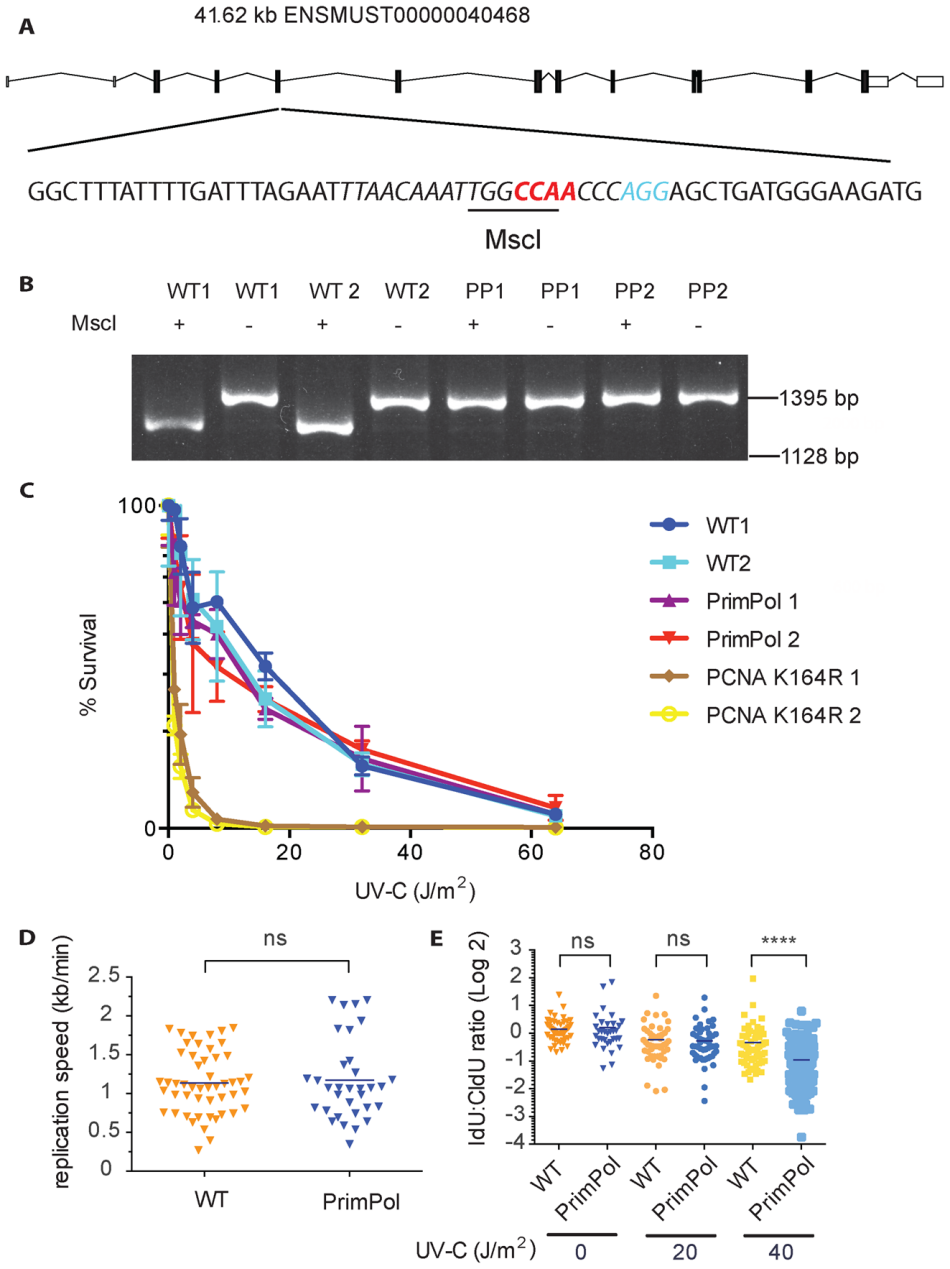
CRISPR/Cas9-mediated inactivation of PrimPol. (**A**) Schematic presentation of the CRISPR/Cas9-based inactivation of *PrimPol* by CRISPR/Cas9 and sgRNA (cursive, PAM in blue). A four-base pair CCAA (red) deletion in exon 5 renders the single mRNA of PrimPol out of frame and destroys the indicated MscI restriction site. (**B**) The *PrimPol* gene has a single mRNA transcript. A nested PCR was performed on the cDNA spanning exon 1–15, followed by a nested primer set spanning exon 3–14. Subsequently, the cDNA was cut using MscI enzyme (Thermo Scientific), to verify the CCAA deletion that destroyed the MscI site in the *PrimPol ^Δ^* allele. (**C**) UV-C sensitivity of *PrimPol* deficient primary pre-B cells. The UV-C sensitivity of wild-type (WT) and *PrimPol* deficient pre-B cells was determined by measuring survival in response to increasing UV-C doses. PCNA^K164R/K164R^ mutant cells were included as positive controls (28). (**D** and **E**) Replication speed and fork progression after UV-C treatment. For DNA fibre assays, MEFs were first labelled with CldU for 20 min and then 20 min with IdU. After CldU, cells were exposed to 20 J/m^2^ or 40 J/m^2^ of UV-C light. The lengths of CldU tracks were measured and the average replication speed (kb/min, error bar indicates standard deviation) on a non-damaged template was measured. (D) For analysis of replication speed of WT and *PrimPol^Δ/Δ^* in absence of DNA damage, we measured the length of the CldU track and calculated the fork speed in kb/min. (E) For analysis of replication progression upon UV-C induced DNA damage, IdU:CldU ratios of mock and UV-C treated WT and *PrimPol^Δ/Δ^* MEFs were calculated. At least 50 DNA fibres were analysed per experiment. A representative experiment is shown (of two). The Mann-Whitney test was applied to determine statistical significance. NS means not significant, **** indicates *P* < 0.0001.

In mice, the *PrimPol* locus locates on chromosome 8 and the spliced transcript comprises 15 exons encoding a single mRNA of 3812 nt (Figure 1A). To define the consequences of a PrimPol deficiency in mice, we took advantage of the CRISPR/Cas9 genome editing technology to inactivate PrimPol (27,32). To minimize potential confounding issues associated with long-term embryonic stem cell (ES) culturing, backcrossing and conventional gene targeting (33,34), we choose for zygote injection (35). Cas9 mRNA and a single guide RNA (sgRNA) for exon 5 of *PrimPol* (ENSMUSE00001229433 at position 362–365 in the ORF of *PrimPol*) was injected in 143 zygotes. Of these, 126 developed into two-cell embryos and were implanted in eight surrogate mothers. In total, 34 pups were born and screened for insertion/deletion mutations. A founder was identified with a 4-nt deletion (CCAA) in exon 5 causing a frame-shift and the deletion of the highly conserved N122 in the catalytic domain (Supplementary Figure S1A). PCR amplification of cDNA from MEFs of *PrimPol* mutants with external primers (spanning exon 1–15) in combination with a nested primer set (spanning exon 3–14) confirmed the presence of a single transcript with the expected 4 nt deletion, which destroys a MscI site in the wild-type allele (Figure 1B). This mutant *PrimPol* allele (*PrimPol^Δ^*) results in a premature stop, giving rise to a truncated polypeptide of 127 amino acids that lacks a critical portion of the archaeo-eukaryotic primase domain and the entire C-terminal Zn-finger regions essential for the priming activity of PrimPol (Supplementary Figure S1A) (25,26). As determined by qPCR the premature stop codon resulted in a 50% reduction of PrimPol mRNA compared to wild-type MEFs (Supplementary Figure S1B). *PrimPol* heterozygous and homozygous mice were viable and born at the expected Mendelian frequency (data not shown).

### UV-survival and replication fork progression in the absence of PrimPol

Primary pre-B cell lines were established and assessed for their sensitivity to UV (Figure 1C). Consistent with a previous knockdown study in human fibroblasts (23), PrimPol deficient and proficient cells displayed equal sensitivity to UV-C induced DNA damage.

To directly determine the impact of UV-C induced lesions on DNA replication speed and progression in a PrimPol deficient condition, we performed DNA fibre assays. The DNA fibre assay allows the visualization of individual replicons on a single molecule level (36,29). Consistent with the concept that PrimPol is a genuine DDT polymerase (24–26), PrimPol was found dispensable in determining the replication speed during unperturbed replication (Figure 1D). This observation is consistent with an independent report by Wan *et al*. (37), however, contrasts a report by Mourón *et al*. (24), suggesting that the lack of PrimPol in unperturbed Hela cells significantly reduces fork rate. Next, we analysed the relevance of PrimPol in maintaining replication progression in the presence of increased UV-C damage. While PrimPol proficient and deficient cells were equally sensitive to UV-C induced DNA damage inflicted by 20 J/m^2^, at 40 J/m^2^ the IdU:CldU ratio was further reduced indicating impaired replication fork progression in the *PrimPol* deficient setting (Figure 1E). A limited redundancy in the remaining DDT system may explain this threshold in revealing the PrimPol deficiency.

### T and B cell development proceeds normally in the absence of PrimPol

To investigate potential effects of a PrimPol deficiency on early B and T cell development in bone marrow and thymus respectively, the cellularity and composition of the B and T cell precursor subsets of *PrimPol^Δ/Δ^* and wild-type litters were compared.

To determine the size of specific B cell precursor subsets in the bone marrow, we distinguished pro-B cells (CD19+, CD45R-low, CD117+, IgM-), pre-B cells (CD19+, CD25+, CD45R-low, CD117-, IgM-), immature B cells (CD19+, CD25-, CD45R-low, CD117-, IgM+) and mature B cells (CD19+, CD45R-high, IgM+), by staining with a cocktail of IgM, CD19, CD25, CD45R and CD117 specific antibodies. Dead cells were excluded from the analysis by propidium iodide staining.

To reveal T cell progenitor subsets in the thymus, single cell suspensions were stained with conjugated antibodies, specific for CD3, CD4, CD8a, CD25, CD44 and TCRβ, to measure the size of the double negative (DN, CD4-/CD8-), double positive (DP, CD4+/CD8+) and single positive compartments (CD4+/CD8- and CD4-/CD8+). Furthermore, the developmental subsets within the CD4-, CD8- (double negative compartment were further distinguished by CD44- and CD25-specific staining to reveal DN 1 (CD44+25-), DN 2 (CD44+, CD25+), DN 3 (CD44-, CD25+) and DN 4 (CD44-, CD25-) subsets.

These analyses revealed no alterations in bone marrow and thymus (Supplementary Figure S2A and B). Likewise, the mature B and T cell compartments in the spleen, as determined by CD4 for helper T cells, CD8 for cytotoxic T cells and CD19 for B cells, and CD19-, CD3- for non B/T cells, were indistinguishable between these genotypes (Supplementary Figure S2C). We conclude that the inactivation of *PrimPol* has no critical impact on the development of B or T cell in the bone marrow, thymus and spleen.

### PrimPol is dispensable during class switch recombination

CSR takes place in G1 of the cell cycle and strongly depends on the formation of AID-induced staggered DSBs in active *Igh* switch regions (10,11). The resulting DSBs enable CSR between the two switch regions, where the intervening DNA is circularized and the recombined DNA ends are re-joined by non-homologous or alternative end joining (38). To investigate whether PrimPol controls CSR, we determined the CSR frequencies of CFSE loaded, naïve B cells (CD43-) isolated from the spleen of *PrimPol^Δ/Δ^* and wild-type littermates. CSR to IgG3 and IgG1 of antigen-inexperienced B cells exposed to LPS alone or LPS and IL4, respectively, were found unaffected (Supplementary Figure S2D). Likewise, proliferation, as measured by CSFE dilution, was indistinguishable between both genotypes (Supplementary Figure S2E). Apparently, PrimPol does not impair the proliferative capacity of B cells and is dispensable for CSR.

### PrimPol exerts a strand-biased anti-mutagenic activity during SHM

**Figure 2.**
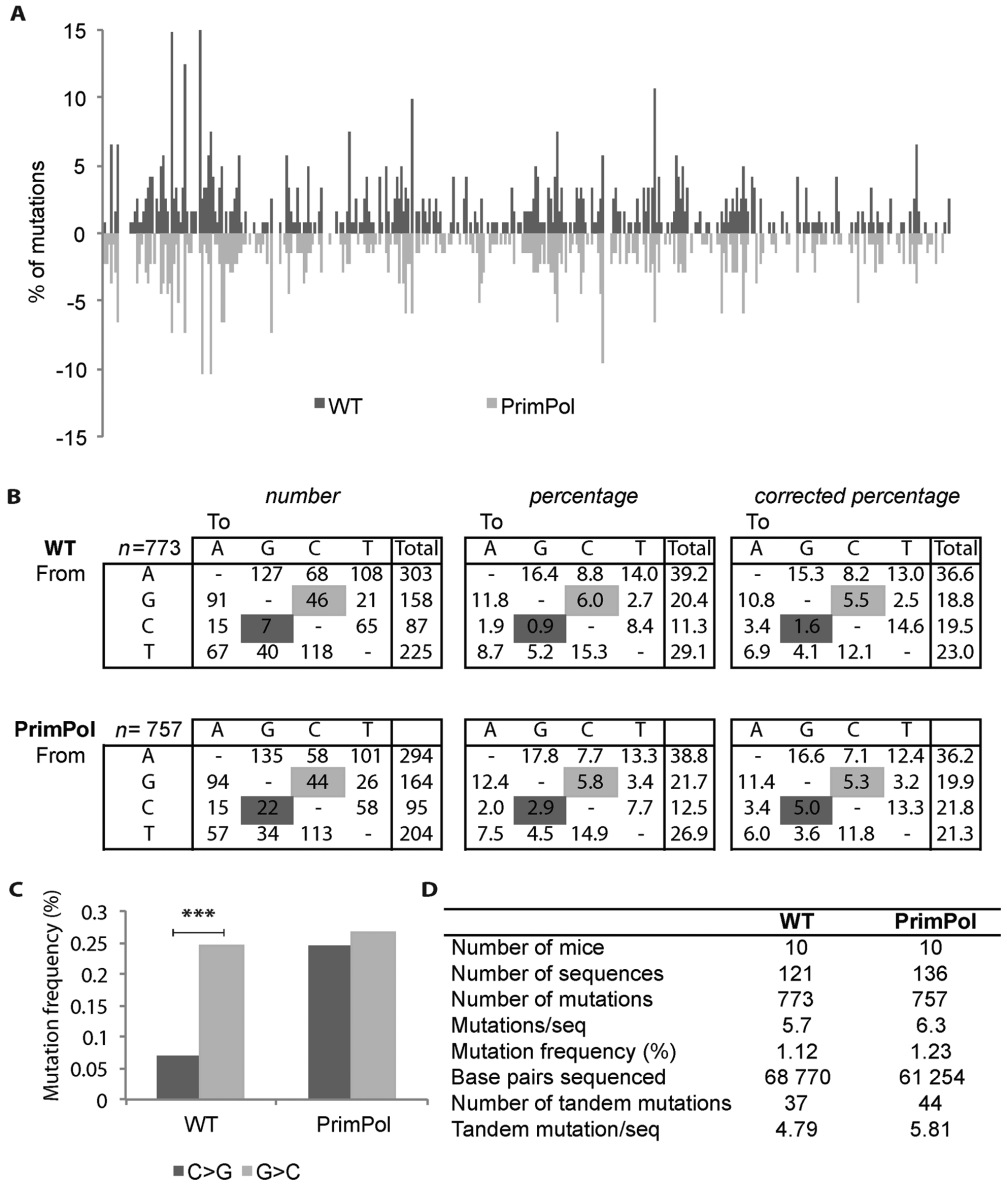
SHM in rearranged J_H_4 intronic sequences from wild-type (WT) and *PrimPol^Δ/Δ^* B cells. (**A**) The distribution of somatic mutations along the J_H_4 intron is displayed as a percentage of mutations per total number of mutated sequences. (**B**) Mutation spectra in hypermutated J_H_4 introns of GC B cells from WT and *PrimPol^Δ/Δ^* mice. The numbers, percentages and percentages corrected for the base pair compositions are depicted. Only mutated sequences were considered. C>G transversions increased compared to WT to a level comparable to G>C transversions in *PrimPol^Δ/Δ^* B cells (χ^2^-test, *P* = 0.0019 significant changes are marked in dark grey (C>G), and G>C is marked in light grey). (**C**) The C>G and G>C transversion bias found in WT is absent in germinal centre B cells of *PrimPol^Δ/Δ^* mice. The asterisk indicates significant changes (χ^2^-test, in WT: *P* = 0.00089, *PrimPol^Δ/Δ^*: *P* = 0.78). (**D**) Data set generation and SHM frequencies in *PrimPol^Δ/Δ^* GC B cells. The number of mice, mutated sequences, base pairs sequenced, number of mutations, tandem mutations and average mutation frequencies are indicated per genotype.

To determine if PrimPol modulates the mutational profile of hypermutated Ig genes, Peyer's patches were collected from the ileum of *PrimPol* deficient and proficient littermates. After isolation of the genomic DNA from sorted wild-type and *PrimPol^Δ/Δ^* germinal centre B cells, the J_H_4 intronic region was amplified by PCR and non-selected mutations were determined as described previously (31). The two SHM data sets were built from mutated sequences of germinal centre B cells from 10 *PrimPol^Δ/Δ^* and 10 wild-type littermate controls and provided a total number of 773 and 757 mutations in 68 770 and 61 254 base pairs, respectively (Figure 2D). The average frequency of point mutations in mutated J_H_4 intron of germinal centre B cells from these mice was comparable: 1.12% in the wild type and 1.24% in the *PrimPol^Δ/Δ^* setting (Figure 2D) and followed a highly similar distribution over the sequenced region and mutation load per sequence (Figure 2A, Supplementary Figure S3A). Likewise, no effects on the frequency of single and tandem mutations were observed (Figure 2D). Due to low numbers, insertions and deletions were not analysed (data not shown).

Most remarkably, when comparing the spectra of nucleotide substitutions a prominent inter-genotypic difference was found (Figure 2B and C, Supplementary Figure S3B). The absence of PrimPol resulted in a selective increase of C>G transversions, leaving other G/C as well as A/T mutations unaffected. Apparently, PrimPol is capable of prohibiting selectively the generation of C>G transversions at AP-sites in the (+) strand, which according to previous studies provides the template for the leading strand synthesis in mature B cells (39–41). A close examination of the G/C mutations in the SHM spectra of wild-type mice revealed a yet uncharacterized 3.4-fold G>C over C>G transversion bias (Figure 2B and C). This finding was consistent with the mutations spectra in the J_H_4 regions described previously (8,28) (Supplementary Figure S3C and D). In contrast, we did not observe a G>C over C>G bias in the *PrimPol^Δ/Δ^* mice. The loss of the bias was due to a selective increase of C>G transversions in the *PrimPol^Δ/Δ^* setting, implicating a selective strand-biased PrimPol activity in tolerating AP-sites on the leading strand (Figure 2B and C).

### PRIMPOL has an anti-mutagenic role in human invasive breast cancer

**Figure 3.**
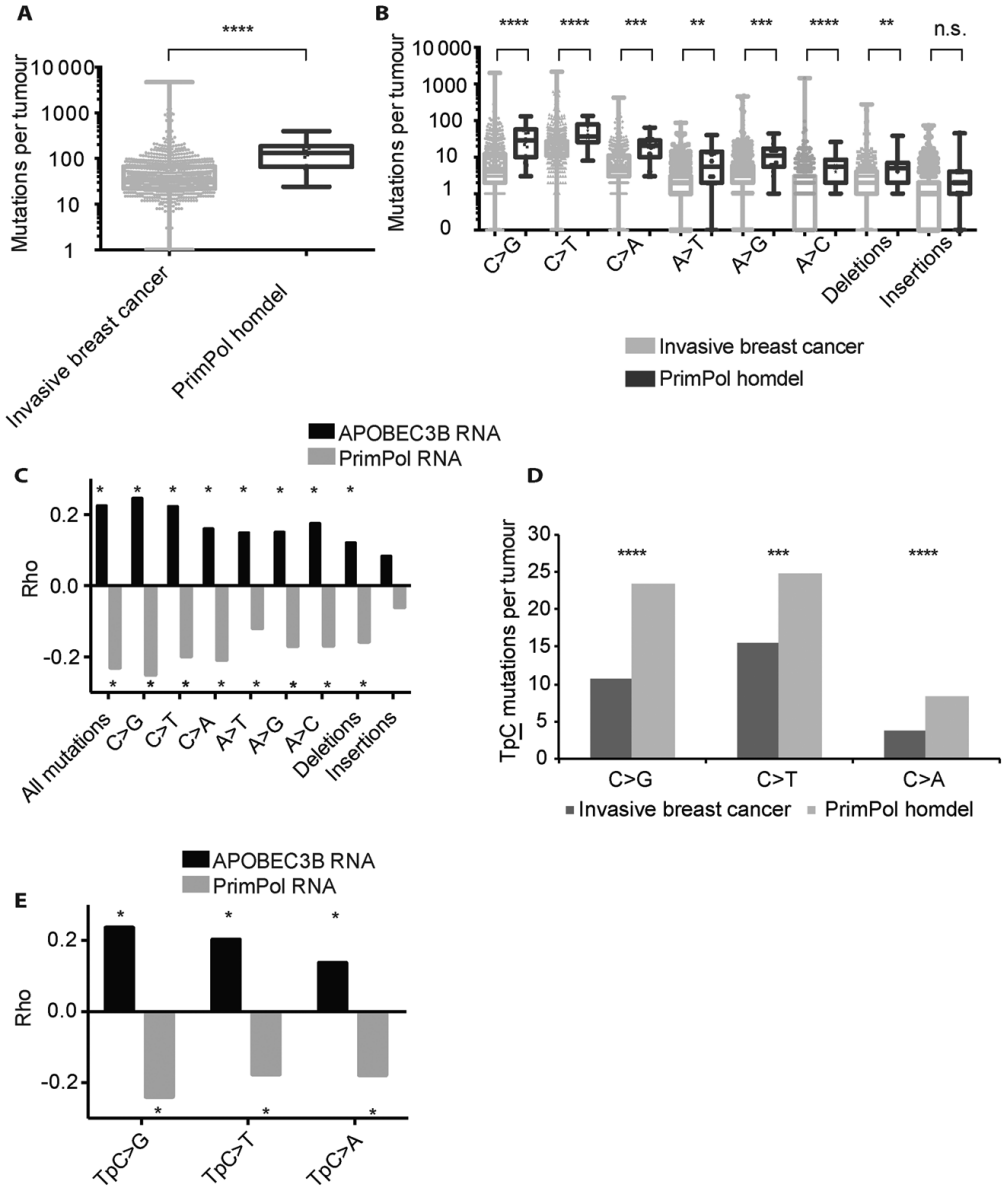
PRIMPOL expression and mutation load in human invasive breast cancers. (**A**) The point mutation load in the exomes of invasive breast cancers compared to the *PRIMPOL* deficient subset. The number of mutations is depicted as log10 scale, boxes contain 50% of all values and bars indicate the highest and lowest values. PrimPol deficient tumours display significantly more point mutations. Asterisks indicate significant differences (*P* > 0.0001). (**B**) Specific base substitutions, insertions and deletions in the exomes of invasive breast cancers and the PRIMPOL deficient subset. The number of mutations is depicted as log10 value, boxes contain 50% of all values and bars indicate the highest and lowest values. Asterisks indicate significant differences. *P*-values as determined by the Mann-Whitney test were as follows: C>G: *P* > 0.0001, C>T: *P* > 0.0001, C>A: *P* > 0.0001, A>T: *P* = 0.0013, A>G: *P* = 0.0003, A>C: *P* > 0.0001, insertions: *P* = 0.057, deletions: *P* = 0.0029. (**C**) Spearman's rank correlation of the *PRIMPOL* expression or *APOBEC3B* expression with defined mutations in 817 human invasive breast cancers. The Spearman's rank correlation was determined for all point mutations as well as specific base substitutions, small insertions and small deletions. The Rho values of the specific correlations are indicated. These analyses suggest a pro-mutagenic activity of APOBEC 3B and an anti-mutagenic activity of PRIMPOL in invasive breast cancer. Stars indicate *P*-value < 0.01, exact *P*-values are depicted in Supplementary Figures S5 and 6. (**D**) APOBEC3B target sequence TpC mutation is increased in *PRIMPOL* deficient tumours. All TpC mutations increase in PRIMPOL deficient tumours, though C>G and C>A increase by ±2.2 times, while C>T transitions increases by 1.6 times. *P*-values as determined by Mann-Whitney test were as follows: C>G: *P* > 0.0001, C>T: *P* > 0.0008, C>A: *P* > 0.0001. (**E**) Spearman's rank correlation of *APOBEC3B* and *PRIMPOL* expression and TpC mutations per tumour. *APOBEC3B* has a positive correlation with TpC mutations. *PRIMPOL* has a negative correlation on all mutation types. Stars indicate *P*-value <0.01, exact *P*-values can be found in Supplementary Figure S11.

The molecular characteristics of *PRIMPOL* and its anti-mutagenic activity during SHM imply a critical genome-wide role for *PRIMPOL* in genome maintenance. To further explore on the anti-mutagenic activity of *PRIMPOL* in an independent system, we first determined a human cancer type that frequently lost the *PRIMPOL* genomic loci. The highest number of *PRIMPOL* deficient tumours was observed in breast cancer patients diagnosed with invasive breast cancer (1.7%; 14 out of 817), consisting of invasive lobular carcinoma or invasive ductal carcinoma, or a mixed group (42). If PRIMPOL exerts a critical anti-mutagenic activity, the overall frequency of point mutations, as determined by exome sequencing, is expected to increase in *PRIMPOL* deficient tumours as compared to the majority of *PRIMPOL* proficient tumours. Indeed, as expected and observed the overall mutation load nearly doubled in *PRIMPOL* deficient tumours (*P*-value<10^−4^, Mann-Whitney test) (Figure 3A). Furthermore, point mutations were significantly increased at G/C and A/T base pairs and deletions in *PRIMPOL* deficient tumours (Figure 3B). The high load of C/G mutations in invasive breast cancer likely reflects damages caused by APOBEC3B-induced U/G mismatches and AP-sites at C/G base pairs as well as other G/C base damages. In summary, these data indicate a critical genome-wide anti-mutagenic activity of PRIMPOL *in vivo*.

As reported before, *APOBEC3B* mRNA expression has a positive correlation with total load of mutations and is associated with an overall poor prognosis (Figure 3C) (13,16,43). We found APOBEC3B expression directly associated with a strong increase in C>G, C>T, C>A as well as mutations at A/T base pairs and deletions (Figure 3C). This suggests that increased *APOBEC3B* can contribute to the entire spectrum of point mutations. A finding consistent with the notion that APOBEC3B-induced U/G mismatches lead to mutations around the initial U/G mismatch. This observation supports the role of mismatch repair in generating mutations in nucleotides flanking cytosine targeted by APOBEC/AID family (8,44–45). These data further support a pro-mutagenic activity of APOBEC3B in invasive breast cancers.

In contrast, extending the same analyses implicated a clear anti-mutagenic activity for PRIMPOL. *PRIMPOL* expression was found to correlate inversely with the mutation load. This observation affected all substitutions and deletions, and was strongest for C>G transversions, and increased 3-fold in *PRIMPOL* deficient tumours. To exclude that gains and losses of the genomic locus of *PRIMPOL* underlies the correlation with mutation load, we extended the analysis to neighbouring genes of *PRIMPOL*. Expression of the neighbouring genes *CASP3* and *CENPU* genes revealed a positive correlation, indicating that the correlation of *PRIMPOL* expression does not depend on gains and losses of the genomic locus (Supplementary Figure S4).

### PRIMPOL prevents APOBEC3B-mediated mutagenesis

APOBEC3B contributes to invasive breast cancer mutagenesis and cytosines within TpC dinucleotides have been identified as preferred APOBEC3B deamination targets (13,16,43,46). Consistent with these notions, we observed that the majority of mutations at C occurred in TpC dinucleotides (54%, 30 out of 56 C mutations per tumour). Most relevant, in *PRIMPOL* deficient tumors these mutations at TpC further increased 2.2-fold for TpC>G and TpC>A transversions and 1.6-fold for C>T transitions (Figure 3D, Supplementary Figure S14).

As an independent approach in assessing the anti-mutagenic activity of PRIMPOL and the pro-mutagenic activity of APOBEC3B, we determined the relation between *PRIMPOL* and *APOBEC3B* expression with the mutation load, specifically TpC>G, TpC>T and TpC>A mutations. Consistent with the pro-mutagenic activity of APOBEC3B, Spearman's correlations revealed that high levels of *APOBEC3B* expression increased TpC>G mutations, but also TpC>T and TpC>A mutations (Figure 3E). As suggested previously, C deamination by APOBEC3B predominantly generates C>T transitions, and in invasive breast cancer this is the most abundant point mutation.

Consistent with the anti-mutagenic activity of PrimPol during SHM, the level of *PRIMPOL* expression correlated negatively with the mutation load in invasive breast cancers. High levels of *PRIMPOL* expression counteracted TpC>G, TpC>T and TpC>A mutations (Figure 3E).

In summary, our analyses reveal an anti-mutagenic activity of PRIMPOL in genome maintenance. Consistent with the anti-mutagenic activity of PrimPol in prohibiting transversions at AID-induced AP-sites in germinal centre B cells, mutation spectra in human invasive breast cancers strongly suggest that this activity also applies to APOBEC3B-induced AP-sites and likely all AP-sites generated during Base Excision Repair.

### Y-family TLS polymerases display anti- and pro-mutagenic activity

**Figure 4.**
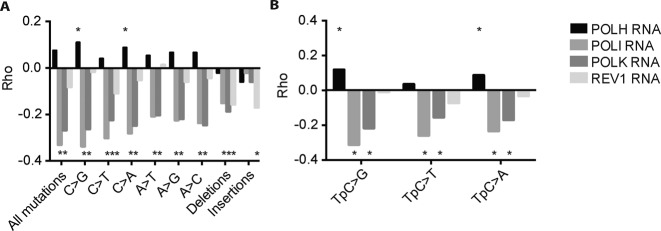
Y-family TLS polymerases correlate both positively and negatively with mutations in invasive breast cancer. (**A**) Spearman's rank correlation of Y-family TLS polymerases with specified mutations. Stars indicate *P*-value <0.01, exact *P*-values can be found in Supplementary Figures S7–10. (**B**) Spearman's rank correlation of Y-family TLS polymerases with TpC mutations per tumour. Stars indicate *P*-value <0.01, exact *P*-values can be found in Supplementary Figures S12 and 13.

While TLS polymerases enable direct replicative bypass of DNA lesions that otherwise stall the replicative DNA polymerases, the activity of TLS can come at the risk of increased mutagenesis (1–4). The question whether in general TLS as component of the DNA damage response network leads to an increased or decreased mutagenesis remains to be addressed. To assess this important issue in an unbiased manner, we also correlated the mutation load (point mutations, deletions and insertions) with the expression level of the Y-family TLS polymerases, *POLH, POLI, POLK* and *REV1*. These analyses revealed that especially *POLK* and *POLI* displayed a strong anti-mutagenic pattern on all types of base substitutions and deletions, indicating a prominent anti-mutagenic role for POLK and POLI in genome maintenance (Figure 4A). In contrast, *REV1* expression level had only a slight anti-mutagenic correlation with C>T mutations, which however was not significant at TpC>T dinucleotides mutations and other base substitutions (Figure 4B). This observation likely relates to a dual pro- and anti-mutagenic role of REV1, which apart from its selective deoxycytidyl transferase activity is capable of recruiting other TLS polymerases (47,48). Interestingly, *REV1* expression does correlate negatively with insertions and deletions, indicating that REV1 likely counteracts the generation of these mutations. Remarkably, a positive correlation was found between *POLH* expression and C transversions but not C transitions. This also accounted for TpC>G and TpC>A mutations, indicating that *POLH* contributes to the increased TpC>G and TpC>A transversions in APOBEC3B expressing invasive breast cancers.

In summary, these observations reveal TLS as an important arm of the DDR network that primarily prevent mutagenesis and therefore contribute to genome maintenance.

## DISCUSSION

PrimPol displays (re-) priming and TLS capabilities, and is thought to have a function during DDT (23–25). These unique properties led us to study the consequences of PrimPol loss of function *in vivo*. Active mutagenesis processes, which are based on cytosine deamination by APOBEC/AID family members, were used as independent readouts to determine DDT activity *in vivo* (8,28,49–53).

SHM is a programmed mutation process initiated by AID. The mutation spectra of Ung2;Msh2 and K164R;Ung2 double mutants revealed an AID footprint, where G/C transitions appear to arise at similar frequencies on both strands (8,45), suggesting there is no strong strand bias at the level of C deamination by AID. Accordingly, AP-sites are expected to arise at similar frequencies on both strands and block replicative polymerases that cannot tolerate AP-sites. In contrast to these notions, the SHM spectra of the J_H_4 intron of rearranged *Igh* revealed a clear biased G>C over C>G mutation. This was lost in *PrimPol* deficient mice, due to increased C>G mutation frequency. *PrimPol* deletion does not affect C>A/G>T transversions. This observation likely relates to the existence of two alternative pathways that generate C transversions. All transversions depend on the U glycosylase Ung2 and a large proportion (∼50%) also on Msh2 (8). Rev1 acts downstream of Ung2, but not downstream the Ung2/Msh2 hybrid pathway (54). Rev1 generates C>G transversions during SHM (51,54) and is likely counteracted by PrimPol to prevent C>G mutations on the leading strand. The TLS polymerase responsible for C>A transversions is not known. However, as there is no G>T over C>A transversion bias, the generation of these transversions may follow a different route.

**Figure 5.**
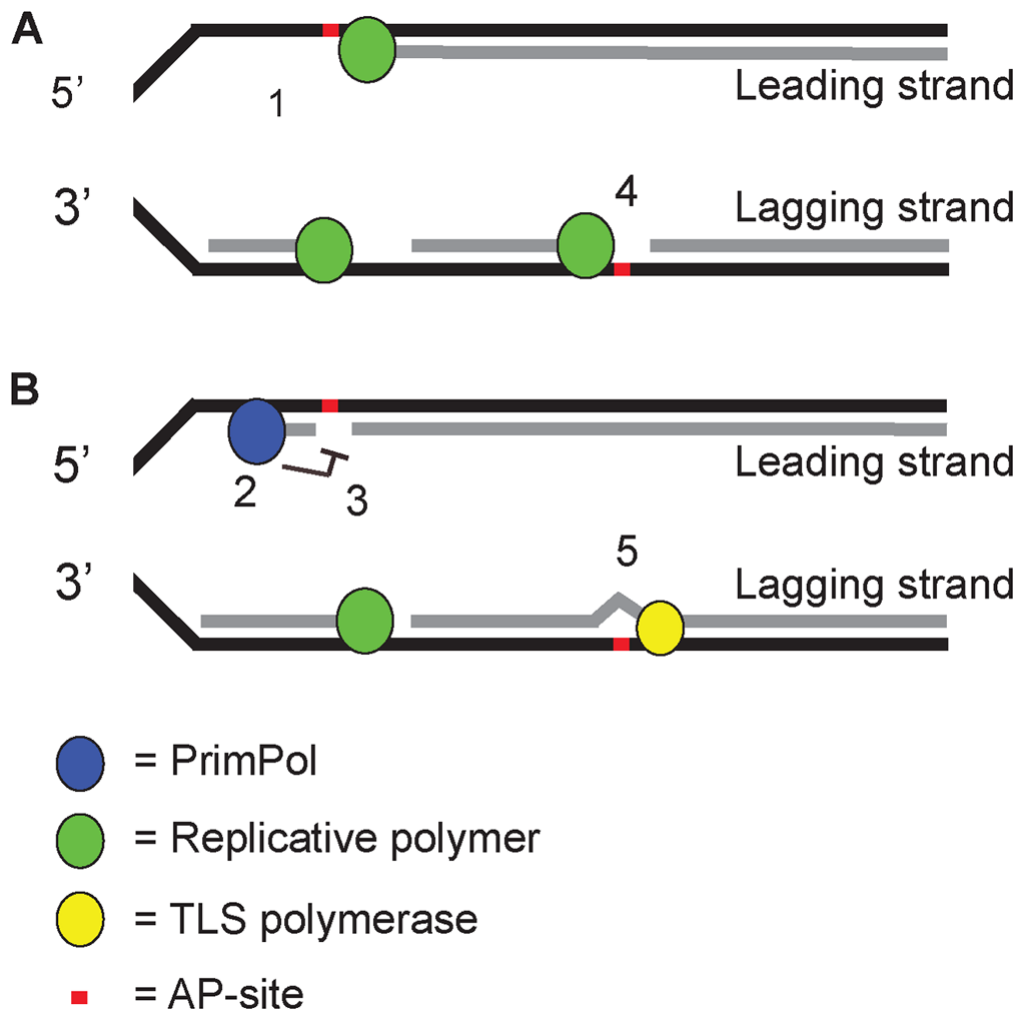
Model: PrimPol prevents mutagenic DDT of AP-sites induced by APOBEC/AID family members. Members of the APOBEC/AID family of cytosine deaminases are associated with programmed mutagenesis. Deamination of C generates a highly mutagenic U, as it instructs a template T. Further processing of U by a uracil glycosylase generates a non-instructive AP-sites that besides C>T transitions can induce C>G and C>A transversions. During SHM, the strand bias regarding G>C over C>G transversions primarily relates to strand-biased PrimPol-dependent error-free DDT of AP-sites on the leading strand. While these findings are based on local SHM spectra in Ig genes, our studies in invasive human breast cancers suggest that PrimPol exerts genome-wide anti-mutagenic DDT activity that likely relates to its repriming capacity on the leading strand. In our model, these AP-sites stall high fidelity replicative DNA polymerases on the leading and lagging strand (1 and 4). Our data imply that PrimPol reprimes downstream of AP-sites in the leading strand to enable fork progression (2) and simultaneously prevents error-prone TLS (3). The remaining gap with the AP-site likely stimulates homology directed error-free repair using the intact template of the sister chromatid. Finally, an AP endonuclease and Polβ restore the remaining AP-site, completing canonical BER initiated by a uracil glycosylase. In contrast, on the lagging strand, repriming of PrimPol at AP-sites does not occur, and AP-sites are tolerated by an AP tolerant TLS polymerase, e.g. Rev1 (5).

As C transversions depend on uracil glycosylase Ung2, these data indicate a strand-biased anti-mutagenic activity of PrimPol at AP-sites, prohibiting C>G transversions downstream of AID and Ung2 in the leading strand. A strand-biased repriming activity of PrimPol is likely responsible in establishing the G>C over C>G strand bias of somatically mutated Igh loci. As G/C transversions are Ung2 dependent, PrimPol likely prohibits mutagenic bypass of AP-site. The strand bias likely relates to an anti-mutagenic activity of PrimPol on the leading strand. This specific C>G transversion effect is consistent with a strong replication origin, which in mature B cells resides around the 3′ regulatory region (39–41). The localization of the replication origin in the 3′ regulatory region places PrimPol anti-mutagenic repriming activity on the leading strand, thereby suppressing C>G transversions (Figure 5). This model where PrimPol acts primarily on the leading strand independently supports a recent report (55). Furthermore, our observations highlight a delicate balance of pro- and anti-mutagenic processing of AID-induced lesions during SHM. In conclusion, the G>C over C>G transversion bias characteristic for hypermutated J_H_4 region is established at the level of strand-biased PrimPol-dependent inhibition of mutagenic TLS. The TLS polymerase responsible for C/G>G/C transversions is likely Rev1, a member of the Y-family of DNA polymerases (51,54).

Apart from AID-induced SHM in rearranged Ig genes, the pro-mutagenic nature of APOBEC3B appears critical in controlling innate immunity of retro-elements. Invasive breast cancers have a high load of APOBEC3B-dependent mutations with a typical TpC dinucleotide signature, suggesting a derailed APOBEC activity during tumourigenesis (16). Here we took advantage of this signature as readout for DDT at APOBEC3B-induced AP-sites in the genomes of invasive breast cancers. Consistent with the anti-mutagenic activity of PrimPol during SHM, the mutation load, including TpC mutations, was significantly increased in the *PRIMPOL* deficient tumour subset. In addition, an inverse correlation between *PRIMPOL* expression and specific point mutations was observed, supporting a critical anti-mutagenic activity and function of PRIMPOL in genome maintenance. The effect of PRIMPOL on mutations at TpC dinucleotides was strongest for C transversions (TpC>G, TpC>A), which is in line with anti-mutagenic activity of PRIMPOL in response to AP-sites generated by APOBEC3B in these tumours.

However, the difference between murine SHM and APOBEC3B mutagenesis in human invasive breast cancer is that PRIMPOL affects C/G transversions and transition in invasive breast cancer, whereas only C>G transversion are affected by PrimPol in our SHM readout. This might be explained by differential processing of AP-sites specific between tissues or species.

In contrast, we observed a strong pro-mutagenic effect of APOBEC3B for all point mutations and deletions, most strongly for C mutations. Similar to the processing of AID-induced DNA lesions during SHM, *APOBEC3B* expression was also found to correlate with mutations at template A/T in invasive breast cancer. These A/T mutations are likely caused by error-prone repair of U–G mismatches, which can involve error-prone TLS polymerase activity even of an undamaged template around the initial U/G mismatch (8,44–45).

As mentioned TLS polymerases enable direct replicative bypass of lesions that otherwise stall the replicative DNA polymerases. In general, TLS is often associated with increased mutagenesis (1–4,56). However, the net effect of TLS polymerase expression on the mutation load has not been determined thus far. Therefore, the question whether in general TLS as a critical component of the DNA damage response network leads to an increased or decreased mutation load remained to be addressed. To approach this important issue in an unbiased manner, we extended our analysis to the entire Y-family of TLS polymerases by comparing the mutation load (point mutations, deletions and insertions) with the expression level of individual Y-family TLS polymerases, *POLH, POLI, POLK* and *REV1*. Our studies revealed an overall anti-mutagenic role for POLI, POLK for all point mutations and deletions, but not insertions. On the contrary, *REV1* expression did except for C>T mutations not significantly correlate with other point mutations. This lack of correlations on point mutations might be due to balanced anti- and pro-mutagenic role of REV1, such as the recruitment of other TLS polymerases and its CMP transferase activity. Interestingly, like POLK and POLI, REV1 appears to prevent deletions, but remarkably was identified as the only Y-family polymerase that prevents insertions. In contrast to all other family members, *POLH* expression showed a positive pro-mutagenic correlation with both C and TpC transversions, suggesting an involvement of POLH in bypassing APOBEC3B-induced AP-sites in invasive breast cancer. This notion is in line with a previous SHM study where in a catalytic-inactive Rev1 setting C>G transversions depended on Polη (57).

Based on these findings we propose a model in which PrimPol exerts a genome-wide anti-mutagenic activity at AP-sites on the leading strand, and likely at other replication blocking lesions. Our model is supported by an independent recent finding, which indicates that APOBEC induced mutations are enriched on the lagging strand (58). We here provide insights into the mechanism underlying these and our observations. While our readouts take advantage of APOBEC/AID family member induced AP-sites, it likely applies to all other AP-sites. On the lagging strand, Polα is the main primase, possibly making repriming by PrimPol obsolete on this strand. Similar to TLS, repriming by PrimPol allows replication to continue effectively. To explain the anti-mutagenic activity of PrimPol, we propose that the remaining gap is either restored by homology directed repair and subsequently canonical repair of the fork-stalling lesion, or a PrimPol-dependent error-free TLS process. However, since AP-sites are non-instructive, it is unlikely that TLS would suddenly become error-free after repriming of PrimPol. Therefore, an error-free repair of the remaining gap by homology directed repair is likely responsible for the anti-mutagenic activity of PrimPol. After homology directed repair, base excision repair can restore the AP-site.

## Supplementary Material

Supplementary Data

SUPPLEMENTARY DATA
