# Supplementary material for: PrimPol prevents APOBEC/AID family mediated DNA mutagenesis
Source: Nucleic Acids Res. 2016 Feb 28;44(10):4734–44. doi: 10.1093/nar/gkw123 (PMC4889928; doi:10.1093/nar/gkw123)
Supplement: Supplementary Data [file gkw123_Supplementary_Data.zip › nar-00164-d-2016-File007.pdf]

Supplementary Material

PrimPol prevents APOBEC/AID family mediated DNA mutagenesis

Bas Pilzecker, Olimpia Alessandra Buoninfante, Colin Pritchard, Olga S. Blomberg, Ivo J. Huijbers, Paul C.M. van den Berk, and Heinz Jacobs

Supplementary Figure 1

(A) Predicted truncated protein of *PrimPol*$^{\Delta/\Delta}$. The truncated protein lacks both, the catalytic archaeo-eukaryotic primase domains (underlined in black) as well as the Zn-fingers (underlined in red) required for repriming.

(B) qPCR for the PrimPol RNA of WT and *PrimPol*$^{\Delta/\Delta}$ MEF's. cDNA was synthesized using random hexamers. Normalization was performed to GAPDH RNA levels (**** indicates p-value < 0.0001).

Supplementary Figure 2. Lymphoid development, proliferative capacity and CSR competence of *PrimPol*$^{\Delta/\Delta}$ B cells

(A) Percentage of progenitor B cell subsets in the bone marrow of wild type (WT) and *PrimPol*$^{\Delta/\Delta}$ mice. A representative example of one of two experiments is shown. Note, no intergenotypic differences were found in the absolute number of bone marrow cells.

(B) Percentage of progenitor T cell subsets in the thymus of wild type (WT) and *PrimPol*$^{\Delta/\Delta}$ mice. A representative example of one of two experiments is shown. Note, no intergenotypic differences were found in the absolute number of thymocytes.

(C) Percentage of mature lymphoid subsets in the spleen of wild type (WT) and *PrimPol*$^{\Delta/\Delta}$ mice. Again, no intergenotypic differences were observed in the absolute number of splenocytes.

(D) CSR to IgG3 and IgG1 was induced in naïve splenic B cells of wild type and *PrimPol*$^{\Delta/\Delta}$ mice by exposure to LPS and LPS/rIL-4, respectively. The frequencies of class switched IgG1 and IgG3 expressing B cells after four days of culture are depicted. PrimPol appears dispensable in determining the CSR efficiency.

(E) The proliferative capacity of *PrimPol*$^{\Delta/\Delta}$ and wild type control B cells was determined by comparing the CFSE dilution profiles. Absence of PrimPol had no impact on cell proliferation.

Supplementary Figure 3.

(A) The mutation load is provided as a frequency of mutated sequences harbouring a defined number of mutations. The genotypes are indicated. The number of mice analysed is indicated in Figure 2D.

(B) Absolute mutation frequency of individual nucleotide substitutions. Values are expressed as the percentage of defined nucleotide substitutions (X>Y) in relation to all nucleotides (X) in the mutated sequences derived from a specific genotype p-value = 0,0019.

(C & D) G>C over C>G bias observed in the JH4 intronic region of hypermutated Ig genes from B cells isolated from C57Bl/6 mice. Data adapted from (8,40), p-values: 0,003 and 0,0009, respectively.

Supplementary Figure 4. Spearman's rank correlations of *CENPU* RNA and *CASP3* RNA with all point mutations. P-value (p) and Rho (r) are indicated.

Supplementary Figure 5: Spearman's rank correlations of *APOBEC3B* RNA with mutations. P-value (p) and Rho (r) are indicated.

Supplementary Figure 6: Spearman's rank correlations of *PRIMPOL* RNA with number of mutations per tumour. P-value (p) and Rho (r) are indicated.

Supplementary Figure 7: Spearman's rank correlations of *POLH* RNA with number of mutations per tumour. P-value (p) and Rho (r) are indicated.

Supplementary Figure 8: Spearman's rank correlations of *POLI* RNA with number of mutations per tumour. P-value (p) and Rho (r) are indicated.

Supplementary Figure 9: Spearman's rank correlations of *POLK* RNA with number of mutations per tumour. P-value (p) and Rho (r) are indicated.

Supplementary Figure 10: Spearman's rank correlations of *REV1* RNA with number of mutations per tumour. P-value (p) and Rho (r) are indicated.

Supplementary Figure 11: Spearman's rank correlations of *APOBEC3B* and *PRIMPOL* RNA with number of TpC mutations per tumour. P-value (p) and Rho (r) are indicated.

Supplementary Figure 12: Spearman's rank correlations of *POLH* and *POLI* RNA with number of TpC mutations per tumour. P-value (p) and Rho (r) are indicated.

Supplementary Figure 13: Spearman's rank correlations of *POLK* and *REV1* RNA with number of TpC mutations per tumour. P-value (p) and Rho (r) are indicated.

Supplementary Figure 14: Analysis of mutation C mutation -1 nucleotide composition.

A

```
Primpol          MLRKWEARVKQIEERASHYERKPLSSVYRPRLAKPEEPSSIWKLFHRQNQAFNFVKSCKE 60
Primpol_CCAA     MLRKWEARVKQIEERASHYERKPLSSVYRPRLAKPEEPSSIWKLFHRQNQAFNFVKSCKE 60
                 ************************************************************

Primpol          SVHVFALECKRGNGQRIYLVTSYAQLWFYYKTRKTLLHCYEVIPENAVCKLYFDLEFNKL 120
Primpol_CCAA     SVHVFALECKRGNGQRIYLVTSYAQLWFYYKTRKTLLHCYEVIPENAVCKLYFDLEFNKL 120
                 ************************************************************

Primpol          ANPGADGKMMVALLIQHVCKALEEFYNVQCSAEDVFNLDSSTEEKFSRHLIFQLHNVAFK 180
Primpol_CCAA     A-----------------------------------------QELMGR------------ 127
                 *                                         :* :.*

Primpol          DNRHAGNFVRKILQPALHLIAEDDEAKVPEAVGQDASGFSVTPLKQEISEAREKVGLPKQ 240
Primpol_CCAA     ------------------------------------------------------------

Primpol          CDPDLSFLVVKNHMETGEKCLFVDLGVYTKNRNFRLYQSSKIGKCVSLEVAEDNRFIPKQ 300
Primpol_CCAA     ------------------------------------------------------------

Primpol          SKDISEENQYFLSSLVSNVRFSDTLRVLTCHPSQTKRKRAECFNSTGTSVESIEGFQGSP 360
Primpol_CCAA     ------------------------------------------------------------

Primpol          YPEVDQFVLSLVNKHDIKGGIRRWNYFFPEELLVYDICKYRWCENIGRAHKSNNIMILVD 420
Primpol_CCAA     ------------------------------------------------------------

Primpol          LKNEVWYQKCHDPVCKAQNFKSTCSPLPTEVSLLFLLKDEDFTSGETDDTSTSLTKDSQT 480
Primpol_CCAA     ------------------------------------------------------------

Primpol          PPSCNLSAGGLSAAAWDDEDDALFLEATEDAEFADAADKSLGSMDDIPDELIIEALQNS 539
Primpol_CCAA     -----------------------------------------------------------
```

B

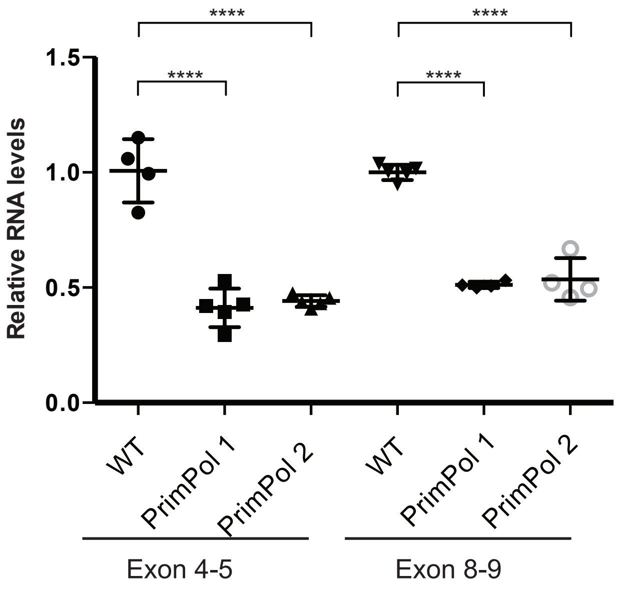

Supplementary Figure 1 Pilzecker et al. 2015

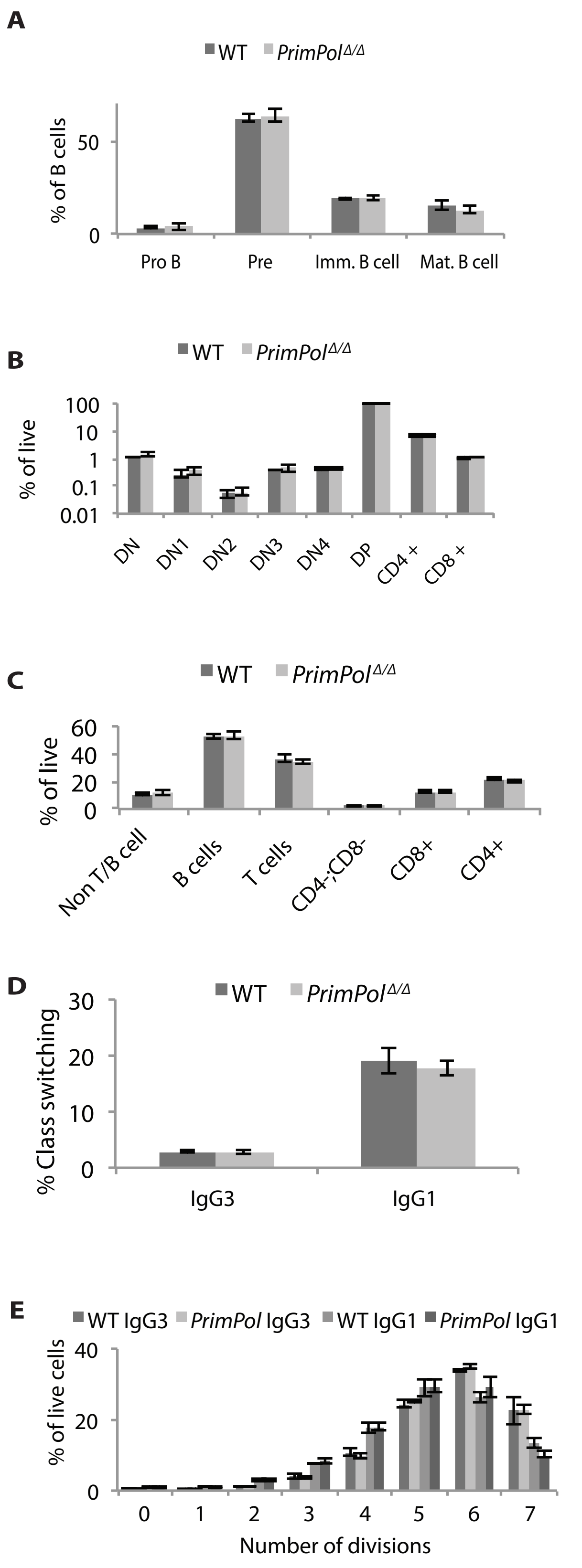

Supplementary Figure 2 Pilzecker et al. 2015

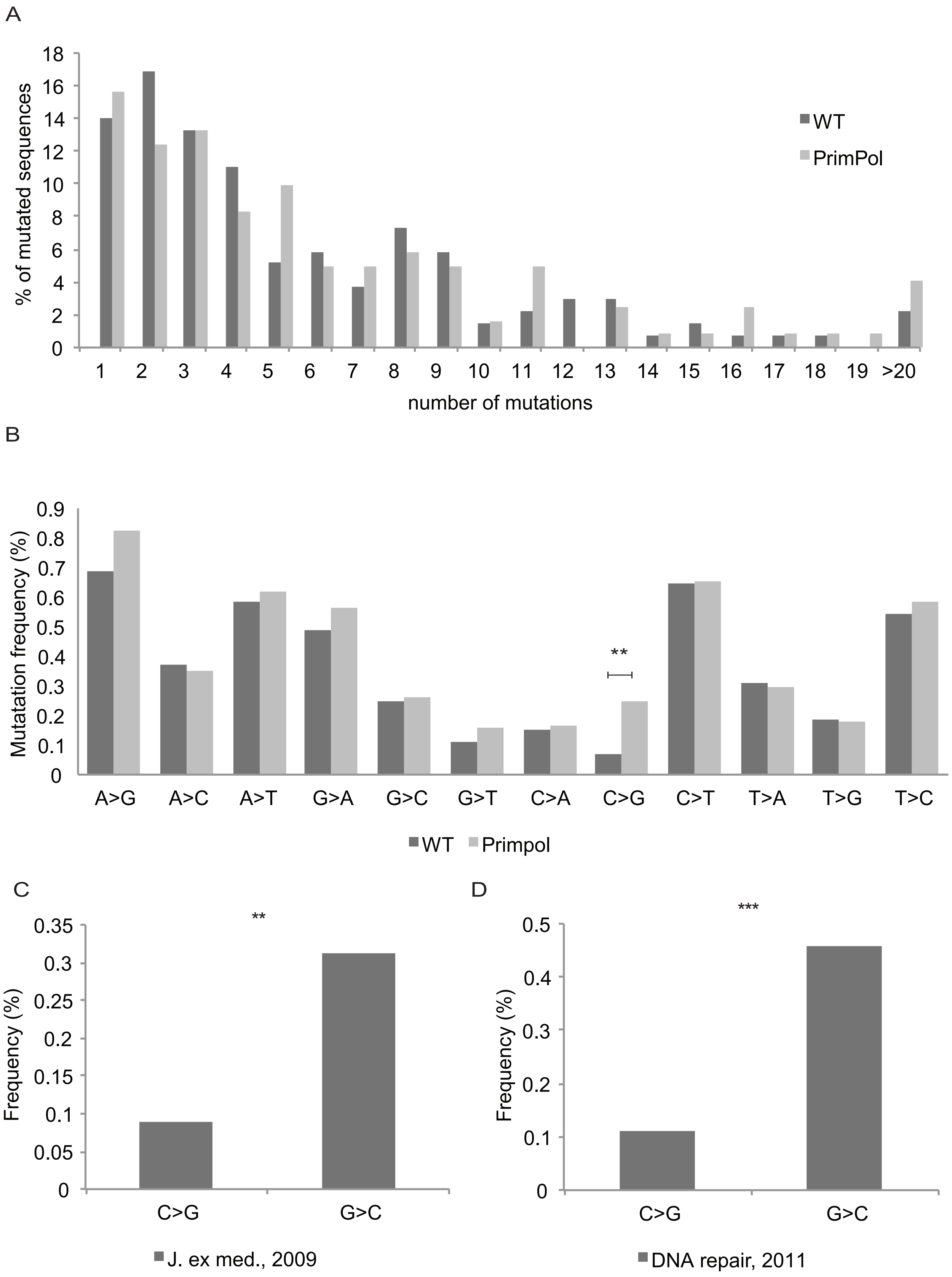

Supplementary Figure 3 Pilzecker et al. 2015

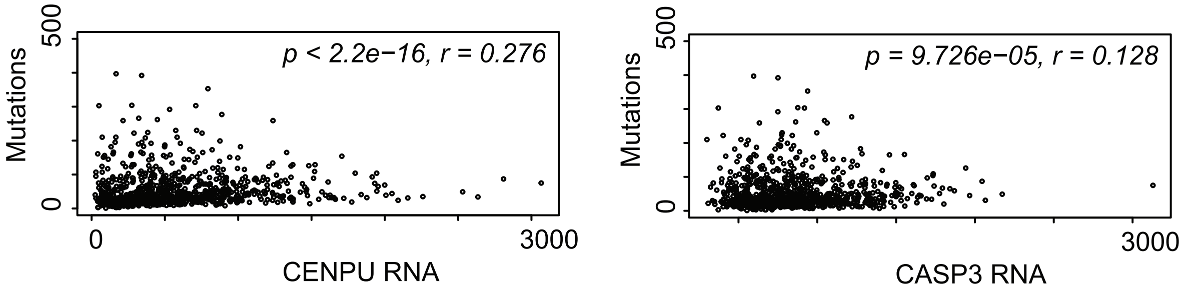

Supplementary Figure 4 Pilzecker et al. 2015

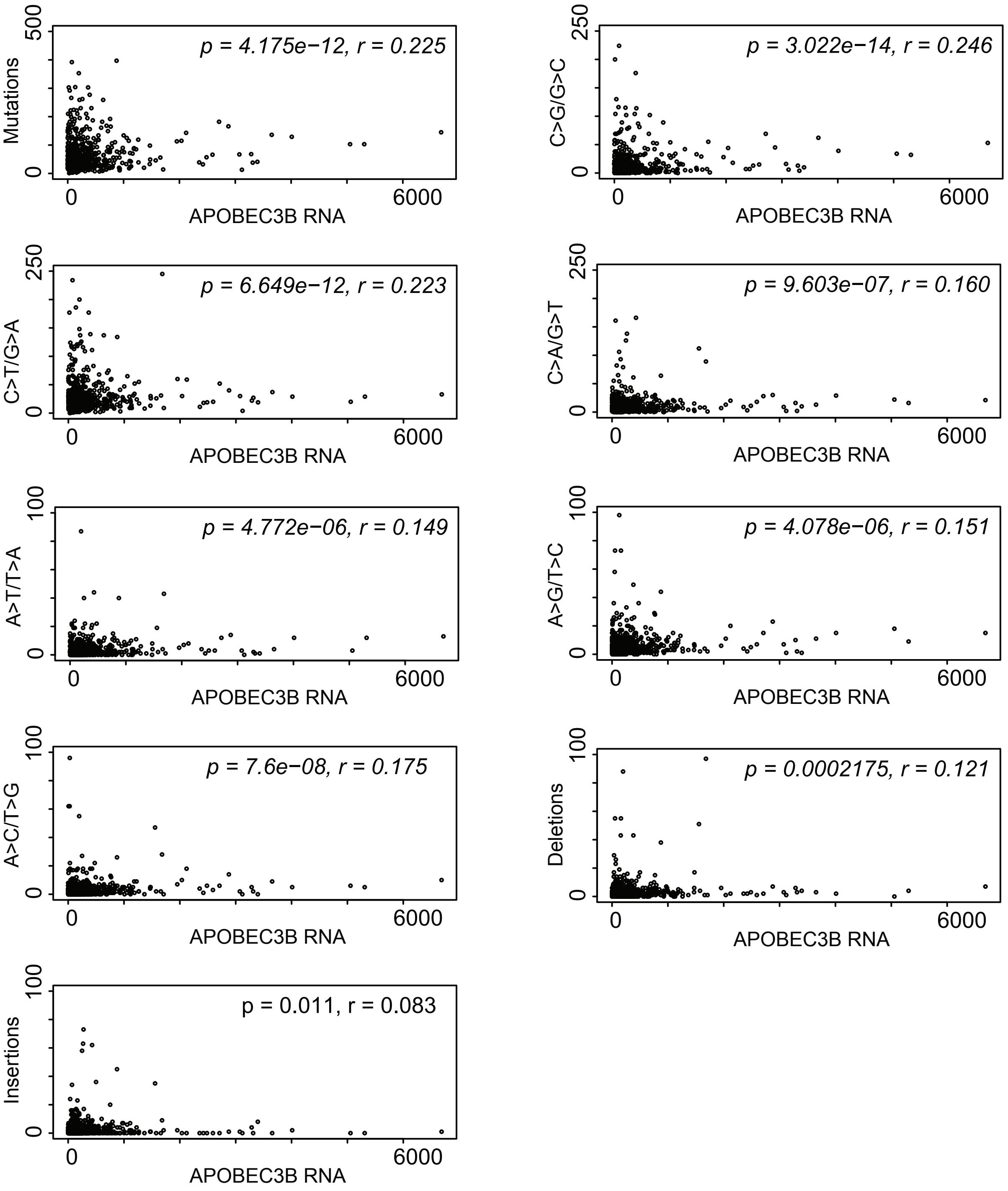

Supplementary Figure 5 Pilzecker et al. 2015

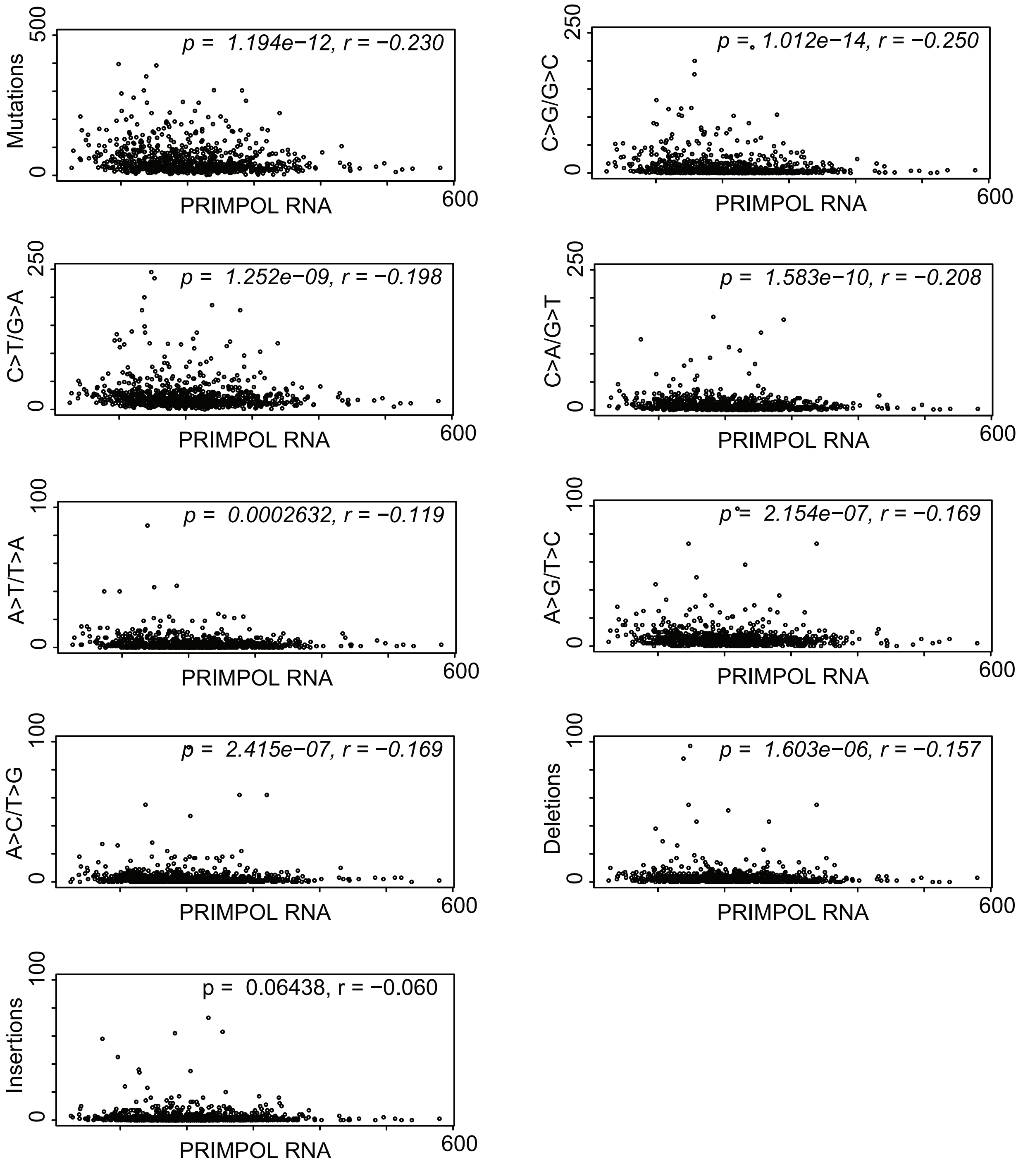

Supplementary Figure 6 Pilzecker et al. 2015

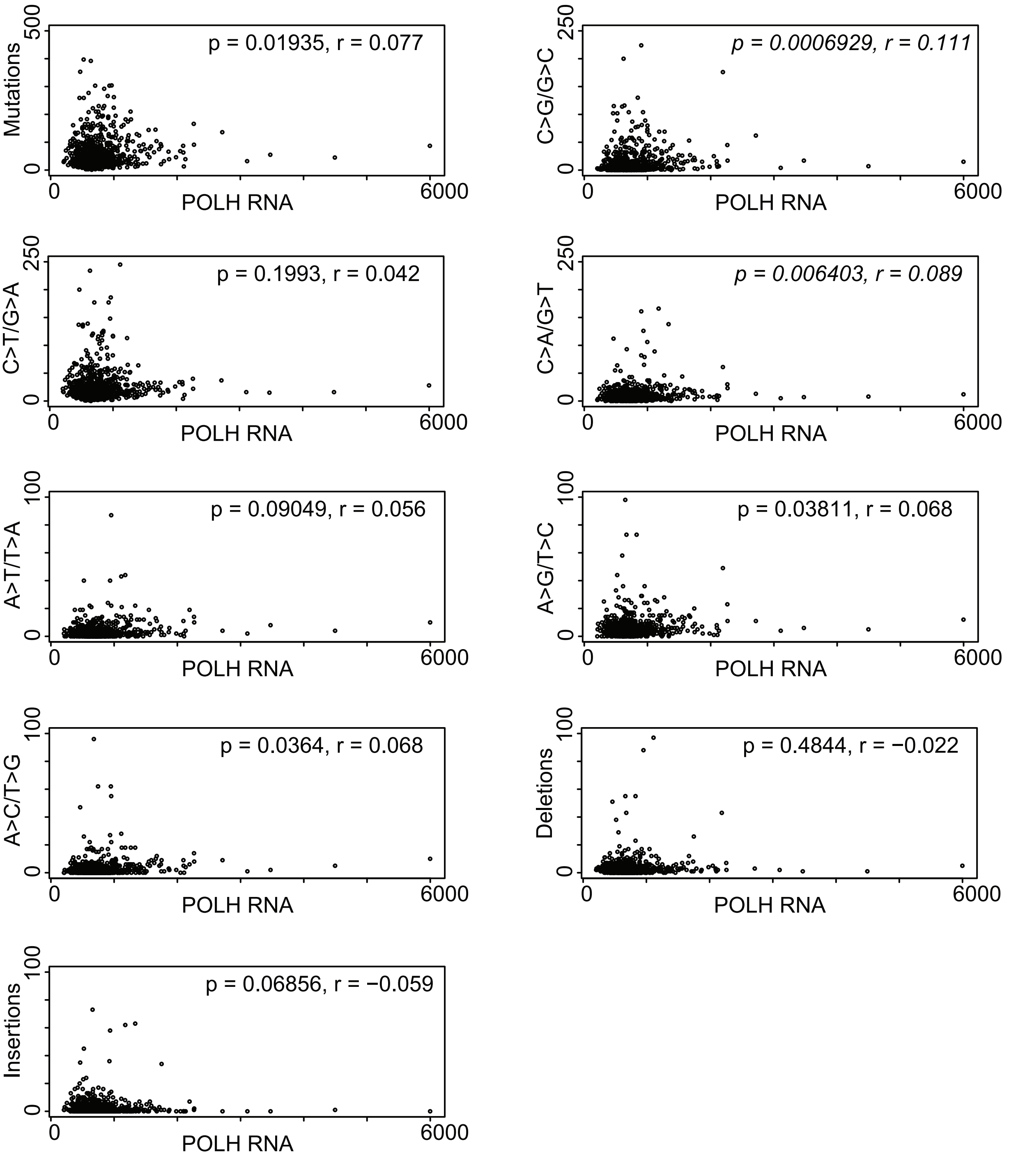

Supplementary Figure 7 Pilzecker et al. 2015

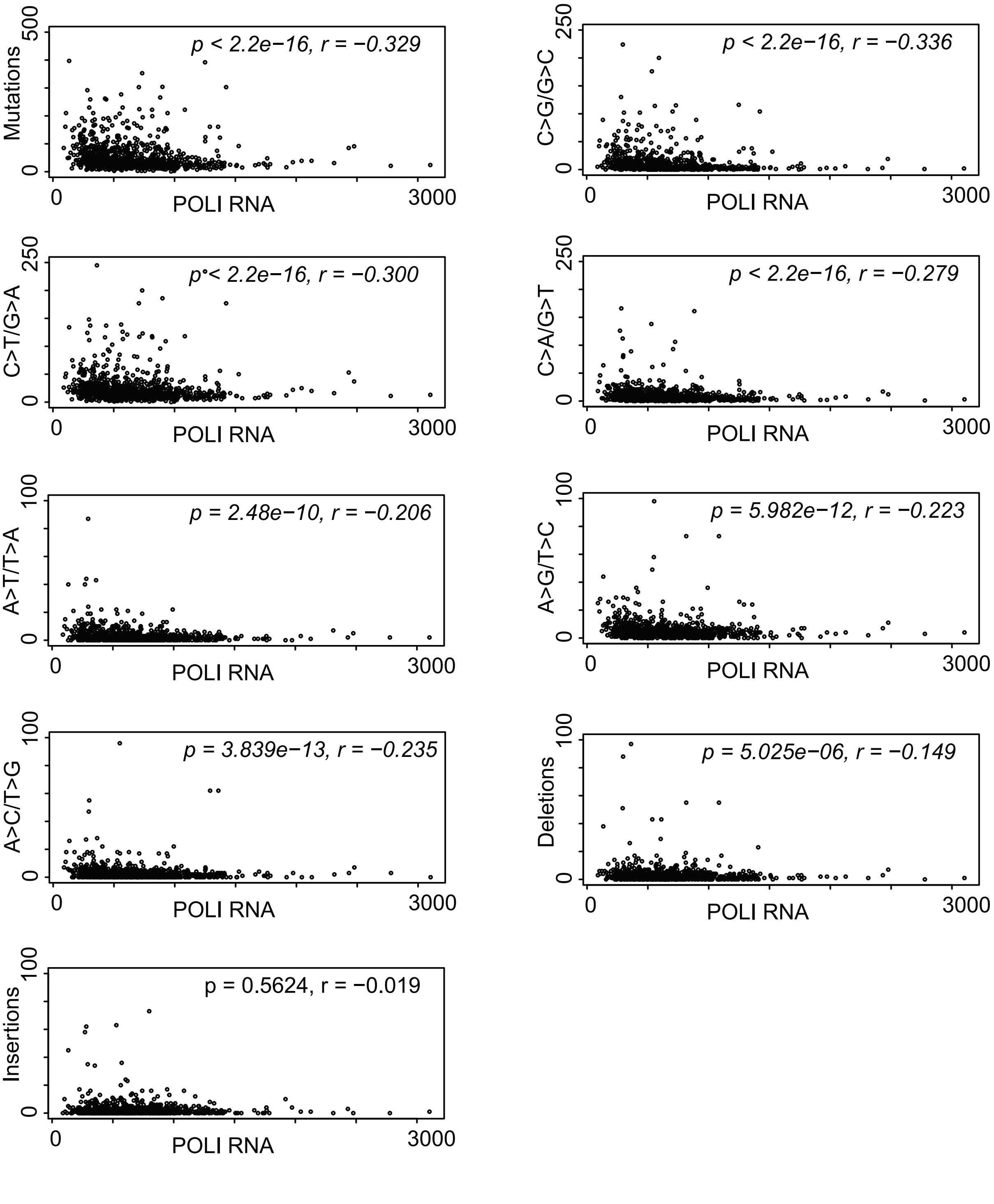

Supplementary Figure 8 Pilzecker et al. 2015

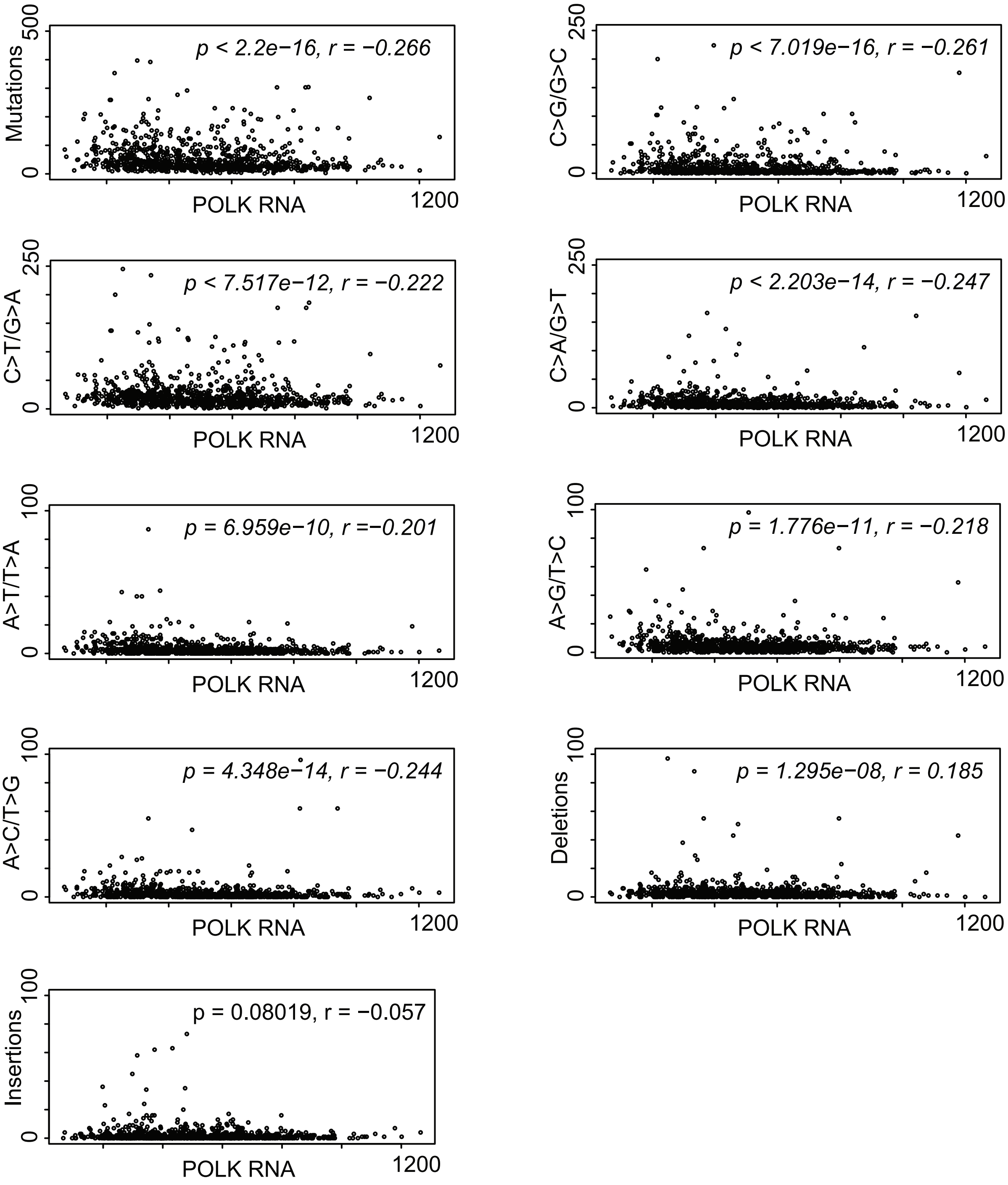

Supplementary Figure 9 Pilzecker et al. 2015

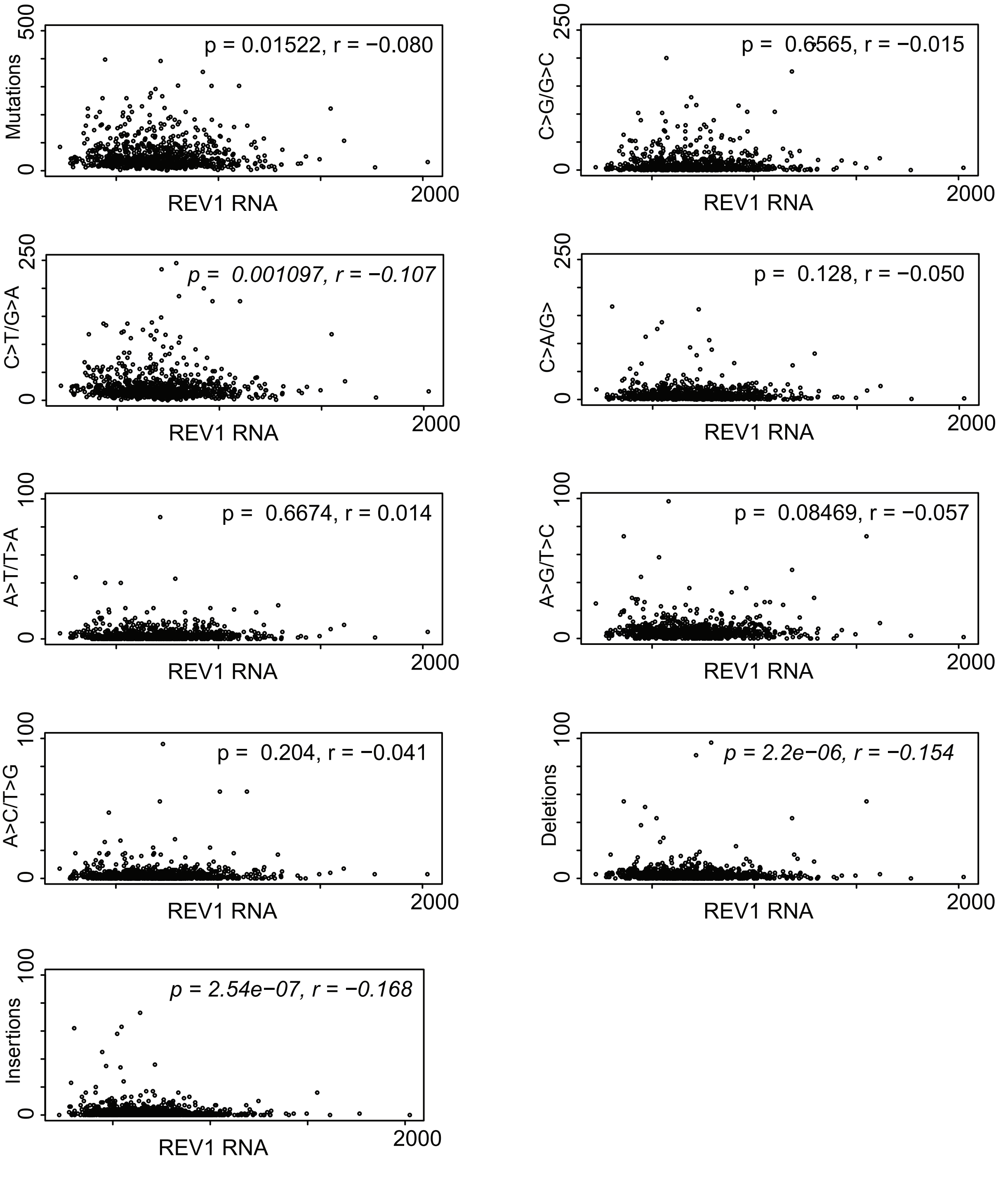

Supplementary Figure 10 Pilzecker et al. 2015

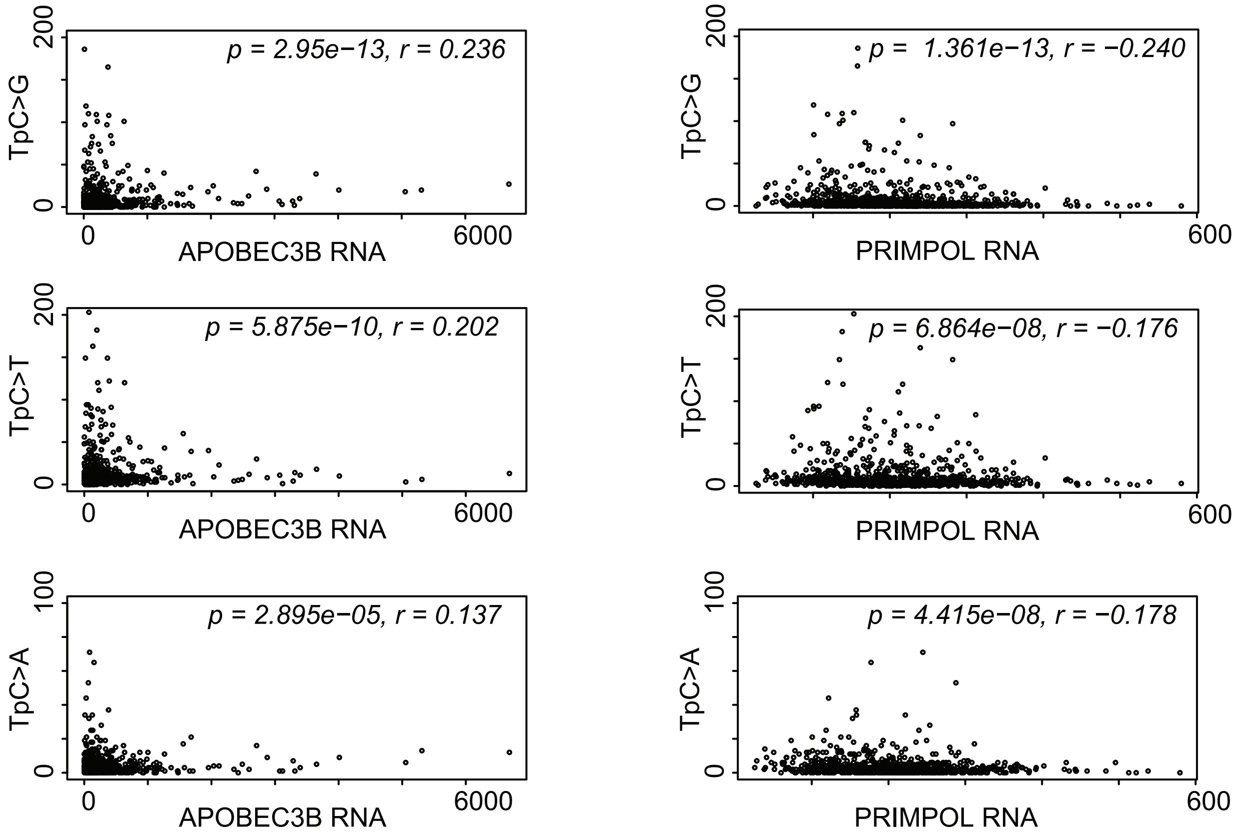

Supplementary Figure 11 Pilzecker et al. 2015

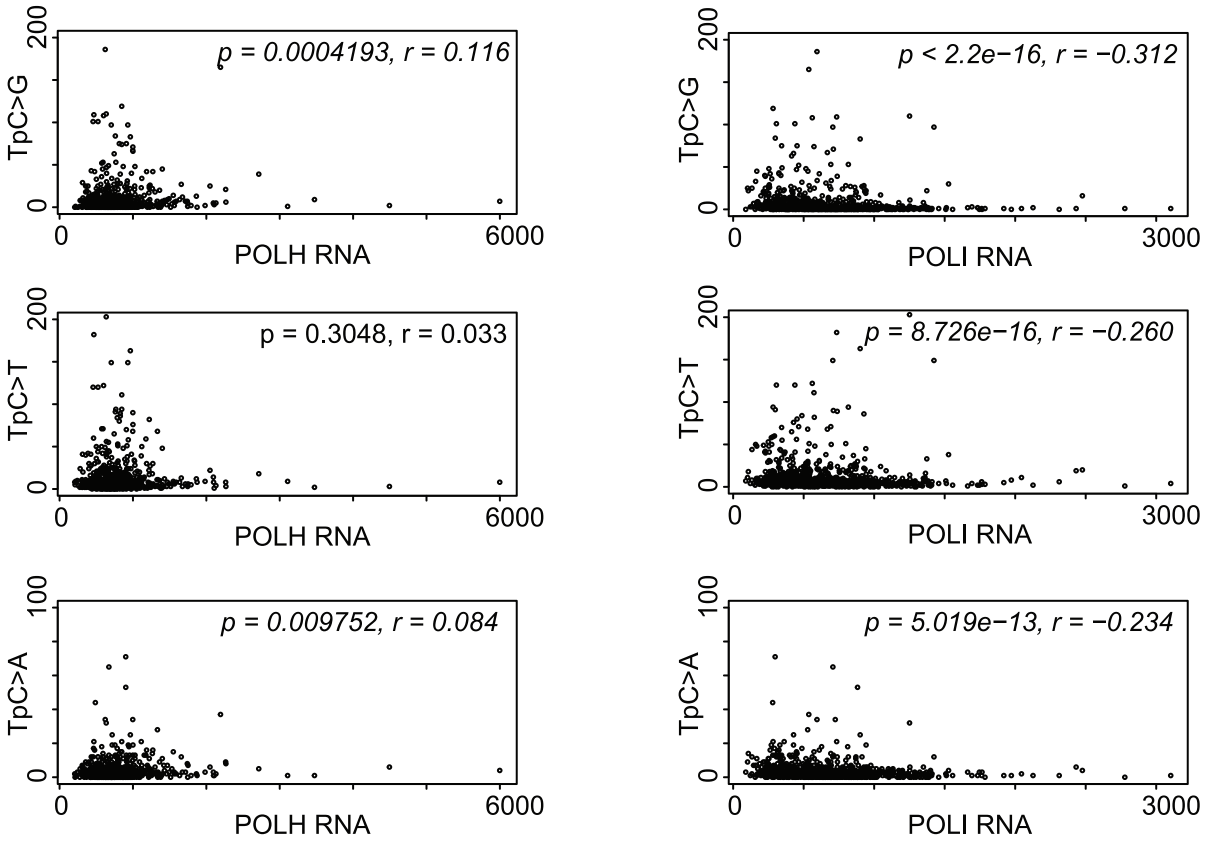

Supplementary Figure 12 Pilzecker et al. 2015

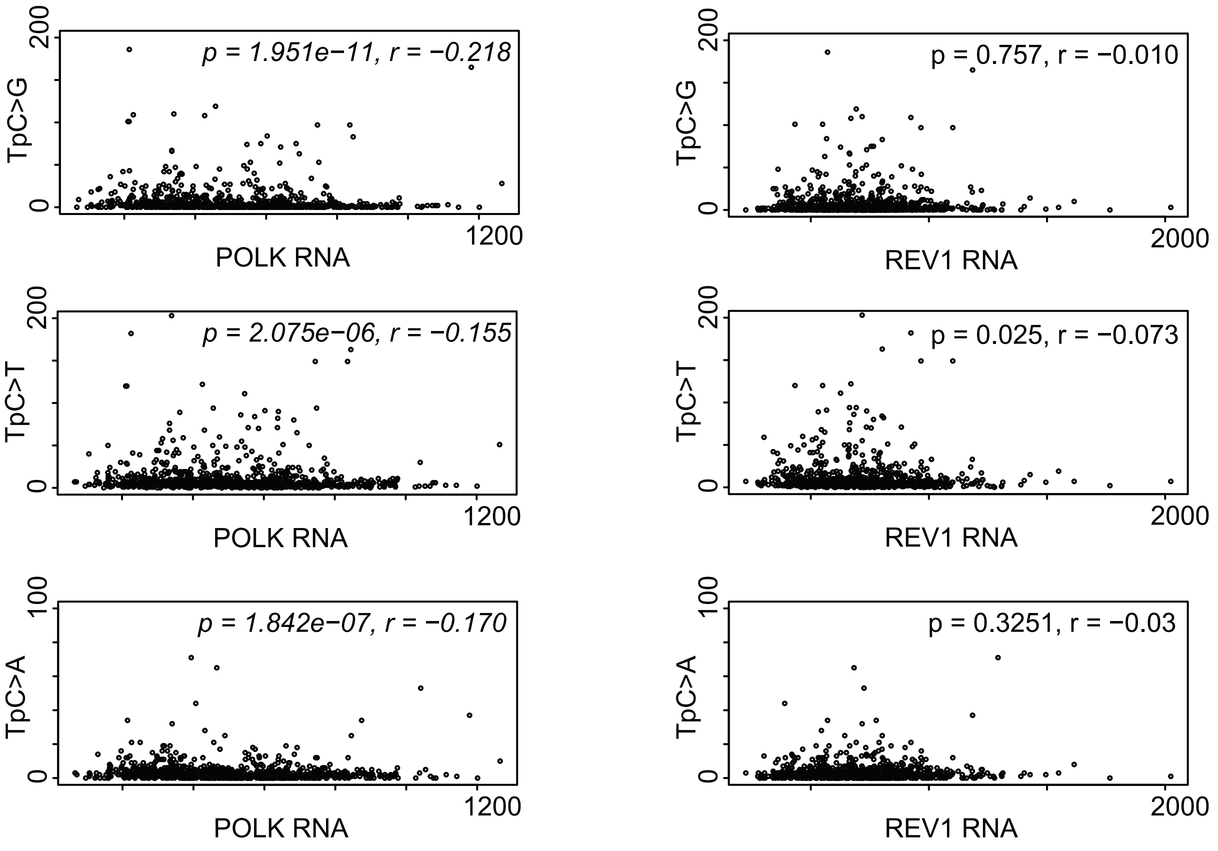

Supplementary Figure 13 Pilzecker et al. 2015

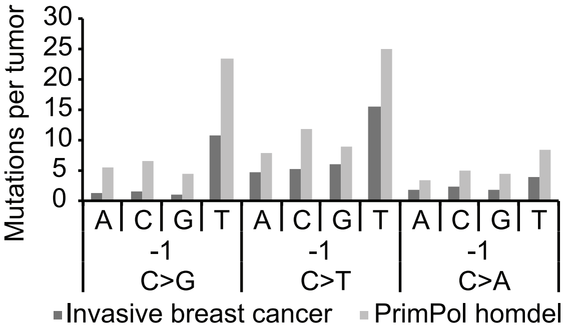

Supplementary Figure 14 Pilzecker et al. 2015
