# Supplementary material for: PrimPol prevents APOBEC/AID family mediated DNA mutagenesis
Source: Nucleic Acids Res. 2016 Feb 28;44(10):4734–44. doi: 10.1093/nar/gkw123 (PMC4889928; doi:10.1093/nar/gkw123)
Supplement: SUPPLEMENTARY DATA [file supp_44_10_4734__index.html]

PrimPol prevents APOBEC/AID family mediated DNA mutagenesis — SUPPLEMENTARY DATA 

# PrimPol prevents APOBEC/AID family mediated DNA mutagenesis

## SUPPLEMENTARY DATA

- SUPPLEMENTARY DATA
